# Dissolution of metal oxides in task-specific ionic liquid†

**DOI:** 10.1039/c9ra06423k

**Published:** 2019-09-19

**Authors:** Janine Richter, Michael Ruck

**Affiliations:** Faculty of Chemistry and Food Chemistry, Technische Universität Dresden 01062 Dresden Germany michael.ruck@tu-dresden.de; Max Planck Institute for Chemical Physics of Solids 01187 Dresden Germany

## Abstract

Due to their typically low reactivity, the activation of metal oxides, as found in ores, earths and minerals, is in general performed by high temperature reactions, which consume much energy. Owing to the prevalence of fossil fuels, this is accompagnied by the generation of large amounts of CO_2_. Alternatively, ionic liquids (ILs) can be used as solvents for metal oxide dissolution and downstream chemistry at much lower temperatures. The dissolution ability of the dry ionic liquid betainium bis(trifluoromethylsulfonyl)imide, [Hbet][NTf_2_], was investigated for 30 metal oxides at 175 °C and compared to chloride containing IL [Hbet]_2_[NTf_2_]Cl. A general dissolution-promoting effect of chloride anions was found, regarding reaction time as well as the variety of dissolved metal oxides. Up to now, the dissolution in [Hbet]_2_[NTf_2_]Cl is limited to basic or amphoteric metal oxides and assumed to be influenced by multiple factors, such as reaction conditions and the lattice energy of the metal oxide as well as its crystal structure. Comprehensive investigations were performed for the dissolution of CuO, which led to the discovery of the water-free complex compound [Cu_2_(bet)_4_(NTf_2_)_2_][NTf_2_]_2_. Proceeding from this compound, a complete exchange of the O-coordination sphere by other ligands was demonstrated, opening up promising possibilities for downstream chemistry.

## Abstract 2

Investigation on the dissolution of 30 metal oxides in the water-free ionic liquid [Hbet][NTf_2_] and the catalytic effect of chloride for the application in green ore processing.

## Graphical abstract

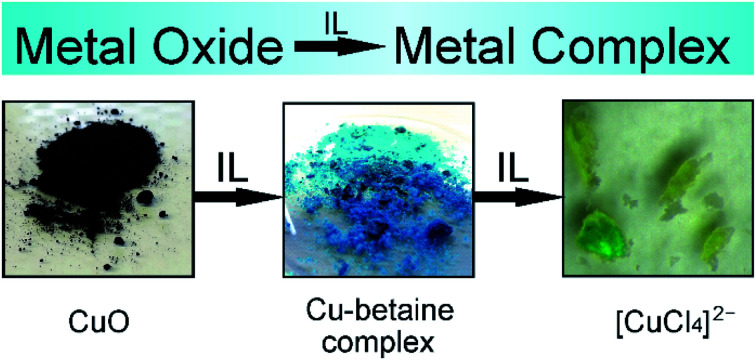

## Introduction

Metals are important resources for many applications, for which reason their extraction from naturally occurring ores, earths and minerals is of great industrial interest. Such natural metal sources often consist of metal oxides alongside concomitant matter (paragenesis). Many of the established metal production processes starting from these resources are based on reactions at high temperatures, typically around 1000 °C, and, thus, are extremely energy consuming and, owing to the prevalence of fossil fuels, CO_2_ producing.^1,2^

Due to the challenges of climate change and the increasing shortage of non-renewable energy resources, a development towards to more sustainable economic processes is of great importance. Innovative, more efficient ways of extracting metal oxides from their natural mixtures of substances and the subsequent production of metals and diverse metal compounds have to be developed.

During the last decades, ionic liquids (ILs) arouse a great deal of interest due to their promising applications to low temperature reactions. ILs, per definition, are salts with a melting point below 100 °C and favourable properties, such as negligible vapour pressure, high thermal stability or a good solubility of numerous inorganic materials.^3^

**Fig. 1:**
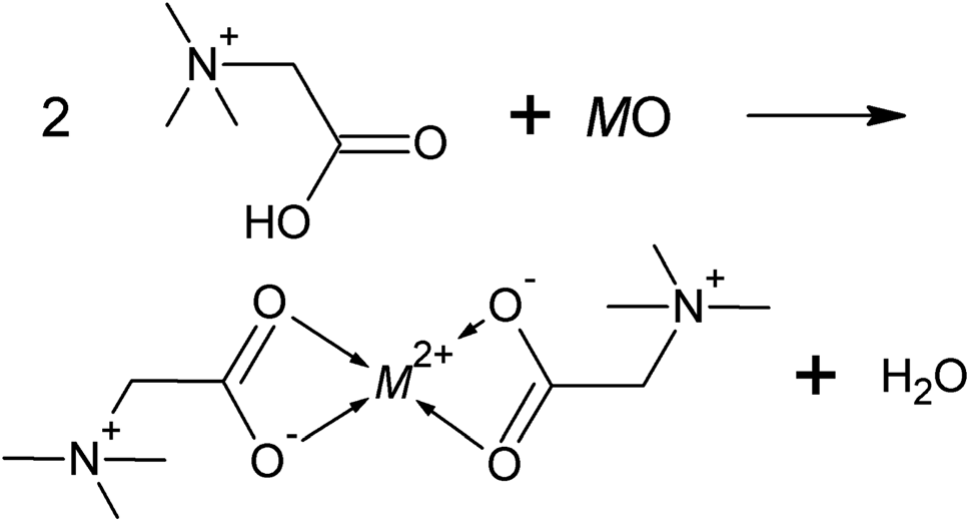
Schematic reaction of a metal oxide and [Hbet][NTf_2_] resulting in a coordination compound and water (M^2+^ = metal ion).

Recently, it was shown that ILs might even be suitable solvents for not readily dissolvable metal oxides. In this context, the presence of coordinating functional groups, as found in so-called task-specific ILs, turned out essential.^4–8^ In the IL betainium bis(trifluoromethylsulfonyl)imide ([Hbet][NTf_2_]), interactions to metal cations occur through the carboxyl group at the cation. In a typical dissolution of a metal oxide, this functional group protonates the oxygen atom of the metal oxide yielding water, while the remaining carboxylate group, subsequently, coordinates to the metal cation,^4^ as illustrated in Fig. 1.

Numerous of such metal–betaine complexes were investigated by Nockemann *et al.*, who also noted the crucial role of water as an additional ligand. Thus, the IL [Hbet][NTf_2_] was merely used as a reactant in an aqueous solution.^4,6^ Other ILs with a slightly varied cation structure, but the carboxyl group still being present, show a similar reaction behaviour with metal oxides.^5^

The possibilities of dissolving metal oxides in as far as possible water-free [Hbet][NTf_2_] was investigated by the group of Binnemans in the course of recycling Nd_2_Fe_14_B magnets. 175 °C was identified as the optimal reaction temperature, avoiding the decompositions of the IL, but supporting reaction kinetics at the same time. A significant increase in reaction time in the absence of water as well as a decrease in the coordination number of the metal ion was reported. This is attributed to the [Hbet]^+^ cation being too sterically demanding to saturate all coordination sites. For this purpose, smaller, additional ligands appear to be necessary, such as water or chloride anions. As demonstrated in a first experiment, the addition of the latter resulted in enhanced dissolution rates.^8^

Based on these findings, in the present work, the dissolution behaviour of various metal oxides in the IL [Hbet][NTf_2_] was investigated. Different from the approach of Nockemann *et al.*^4–6^ but similar to the work of Binnemans,^8^ no additional water was used as this usually involves two major problems regarding downstream chemistry: first, with O-coordinated aqua ligands, a leaching of metal ions from potentially resulting complexes or ligand exchange reactions might prove difficult. Second, the relatively small electrochemical window of water often involves the evolution of oxygen and hydrogen gases in electrolysis. Instead, the complex formation ability of metal oxides with betaine was investigated in pure [Hbet][NTf_2_] and compared to reaction mixtures of the IL and betaine hydrochloride ([Hbet]Cl) as additional ligand source. The long-term goal of these studies is the application to downstream chemistry of metal oxides without the detour over the reduced metal as well as the metal separation from natural sources *via* leaching and subsequent electrolysis. However, the present work is concerned with the initial step of these problems, the dissolution and complex formation of metal oxides in the ILs [Hbet][NTf_2_] and [Hbet]_2_[NTf_2_]Cl.

## Experimental

### Chemicals

[Hbet]Cl (99%), Bi_2_O_3_ (99%), Cr_2_O_3_ (98%), Cu_2_O (99%), CuO (99.9995%), SnO (p.a.) and SrO (99.5%) were purchased from Alfa Aesar. Al_2_O_3_ (p.a.), Nb_2_O_5_ (p.a.) and TiO_2_ (99%) were purchased from Merck. BaO (97%), Fe_2_O_3_ (99%), MgO (p.a.) and V_2_O_5_ (99.5%) were obtained from Riedel de Haën. Ga_2_O_3_ (99.99%), GeO_2_ (99.999%), MoO_3_ (99.999%), Sb_2_O_3_ (99.6%) and V_2_O_3_ (95%) were purchased from ABCR. In_2_O_3_ (99.999%) was obtained from Acros. PbO (99%) and PbO_2_ (p.a.) were purchased from Sigma Aldrich. LiNTf_2_ (80% solution in water) and trihexyltetradecylphosphonium chloride ([P_66614_]Cl, 95%) were purchased from Iolitec. NiO (p.a.) originates from VEB Berlin-Chemie, Co_3_O_4_ (p.a.) from Chempur and CaO (p.a.) as well as WO_3_ (p.a.) from Reachim. MnO (99%) and MnO_2_ (85–90%) were purchased from Sigma-Aldrich. ZnO (p.a.) was obtained from Grillo.

ReO_3_ was synthesised from Re powder (99.5%, Alfa Aesar) by repeated O_2_ addition and subsequent evacuation in a silica ampoule, resulting in Re_2_O_7_. A subsequent chemical transport reaction with I_2_ from 500 °C to 400 °C yielded ReO_3_.

PXRD of ThO_2_ revealed the presence of an unidentified impurity (Fig. S1, ESI†). Similarities with the diffraction pattern of several intermetallic oxide phases suggest (intermediate) products of the ThO_2_ decay chain. **Caution!***ThO*_*2*_*is a weak alpha emitter. All radioactive materials were handled in radioactively controlled facilities that are equipped with personal safety equipment.*

All chemicals were used without any further purification, except for CaO, which was dried at 1000 °C after purchase.

### Synthesis of [Hbet][NTf_2_]

The IL [Hbet][NTf_2_] was synthesized, based on the experimental procedure described in the literature,^4^ by stirring 0.1 mol [Hbet]Cl dissolved in 50 ml water and LiNTf_2_ solution equivalent to 0.1 mol for one hour. Subsequently, the IL was separated and washed with ice cold water until testing with AgNO_3_ solution indicated the absence of chloride ions, typically five times. The IL was dried under vacuum at a rotary evaporator at 60 °C for one hour and at a Schlenk line at 110 °C overnight.

### Dissolution experiments of metal oxides

Dissolution experiments in the pure IL were performed by heating a metal oxide and [Hbet][NTf_2_] in the molar ratio of *n*_M_ : *n*_IL_ = 1 : 4 to 175 °C for 24 h in a system open to the air.

For the reaction of Bi_2_O_3_ and [Hbet][NTf_2_] in the absence of air, the starting materials were weighted in an argon-filled glovebox (M. Braun; *p*(O_2_)/*p*^0^ < 1 ppm, *p*(H_2_O)/*p*^0^ < 1 ppm). The reaction was performed for 24 h at 175 °C under flowing argon gas. After cooling to room temperature, the sample was handled on air.

Chloride containing mixtures were prepared from a metal oxide, [Hbet][NTf_2_] and [Hbet]Cl in the molar ratio of *n*_M_ : *n*_[Hbet][NTf_2_]_ : *n*_[Hbet]Cl_ = 1 : 2 : 2 and heated likewise.

For several metal oxides, mixtures with varying molar ratios or reaction times were investigated. Details are listed in Table S4, ESI.†

Work-up of the CuO samples was performed by the addition of acetone, thus separating CuO. The product yield *η* was determined by weighting the unreacted CuO and using the following formula.
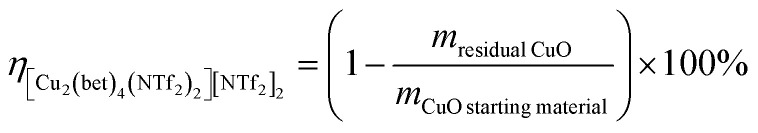


The product could not be obtained phase-pure, as no solvent solely dissolving or not dissolving this complex compound was found. Nevertheless, the product was suitable to perform ligand exchange experiments.

### Calculation of lattice energies and *U*/*x* values

Lattice energies *U* of the metal oxides M_*x*_O_*y*_ were calculated according to literature^9^ with a Born–Haber cycle as phrased in the following equation.
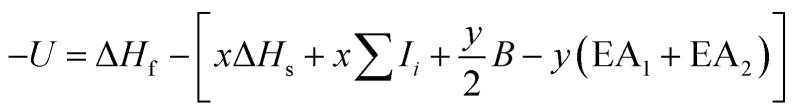
Δ*H*_f_ standard molar enthalpy of formation of M_*x*_O_*y*_, Δ*H*_s_ enthalpy of sublimation of M, *I*_*i*_ ionisation energy (*i* = 1, 2, 3, …), *B* bond enthalpy of O_2_, EA_*i*_ electron affinity of O (*i* = 1, 2).

For metals whose enthalpy of sublimation was not tabulated, the sum of the enthalpy of fusion (Δ*H*_m_) and the enthalpy of vaporisation (Δ*H*_v_) was used. The resulting slight error is <2%.^9^ Furthermore, the ionisation energy of the Co(ii,iii) mixed oxide Co_3_O_4_ was calculated according to 
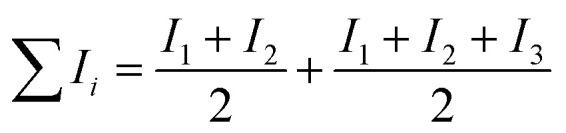
.

*U*/*x* was calculated by dividing the lattice energy by the number of metal atoms in the chemical formula.

Δ*H*_f_ values were obtained from *Thermochemical Data of Pure Substances*,^10^ Δ*H*_s_, Δ*H*_m_, Δ*H*_v_ and *B* from *Lange's Handbook of Chemistry*^11^ and *I*_*i*_ and EA_*i*_ values from the *NIST* online database.^12^

### Ligand exchange experiments

Approximately 50 mg of the reaction product of CuO and [Hbet][NTf_2_] was stirred in 1 g [P_66614_]Cl at 60 °C for 19 h.

### Characterisation

Powder X-ray diffraction (PXRD) was performed on two similarly constructed X'Pert Pro and Empyrean diffractometers (PANanalytical) at 296 K equipped with a curved Ge(111) monochromator in Bragg–Brentano geometry using Cu-Kα_1_ radiation (*λ* = 154.0598 pm). Rietveld refinement was performed with TOPAS^13^ by using the fundamental parameter approach and Le Bail method for modelling preferred orientation.

Infrared (IR) measurements were performed on a Bruker Vertex 70 FTIR spectrometer with attenuated total reflection (ATR) accessory in a radiation range from 500 cm^−1^ to 4000 cm^−1^ and 32 scans per measurement. Data analysis was performed with the programme OPUS.^14^

For Raman spectroscopy, a FT-Raman spectrometer Bruker RFS 100 was used with an excitation wavelength of 1064 nm and 50 to 200 scans per measurement. Due to the strong dispersion of radiation in coloured substances and the resulting high background, additional scans were taken for these samples. Analysis of the data obtained was performed with the programme OPUS.^14^

NMR samples were prepared by dissolving a spatula tip of the samples in deuterated dimethylsulfoxide (DMSO-d_6_) as deuterium lock and filling a NMR tube to a height of approximately 3 cm. ^1^H NMR spectra were recorded on a Bruker Avance WB NMR 300 MHz spectrometer with the resonance frequency 300 MHz.

The collection of diffraction intensities of single-crystals (SCXRD) for crystal structure analysis took place on a single-crystal X-ray diffractometer SuperNova (Rigaku Oxford Diffraction) with Cu-Kα radiation (*λ* = 154.184 pm) at 100 K under flowing nitrogen gas. Empirical multi-scan absorption corrections were applied to the data. Structure solution was performed with the programme SHELXT^15^ in OLEX2 ^16^*via* intrinsic phasing. For structure refinement, the method of full-matrix least squares on *F*_o_^2^ with the programme SHELXL^15^ in OLEX2 ^16^ was used. Non-hydrogen atoms were refined with anisotropic displacement parameters, while hydrogen atoms were refined with riding coordinates and displacement parameters. The crystal structure was plotted in the programme Diamond 4.5.2.^17^ The full crystallographic data is deposited at the Cambridge Crystallographic Data Centre, deposit number CCDC 1947513.†

For energy dispersive X-ray spectroscopy (EDX), a drop of a sample dispersed in acetone was placed on a silicon wafer fixed on a SEM sample holder and stored in a vacuum chamber for three days. The studies were performed on a Hitachi SU8020 scanning electron microscope with an Oxford Silicon Drift Detector (SDD) X-Max^N^. An accelerating voltage of 25 kV was used.

## Results and discussion

### Dissolution of metal oxides in water-free [Hbet][NTf_2_]

After synthesis of the IL, it became apparent from PXRD studies that the IL consists of a mixture of approximately 66% [Hbet][NTf_2_] and 34% [(Hbet)_3_(bet)][NTf_2_]_3_. The powder X-ray diffractograms as well as the Rietveld refinement are shown in Fig. S2 and S3, ESI.† The fact of roughly one sixth of the cations being unprotonated betaine might affect the maximum amount of a metal oxide dissolvable in the IL. However, as the experiments performed in this work are of rather qualitative than quantitative nature, the resulting error is considered insignificant. In the following, the synthesized IL is referred to as [Hbet][NTf_2_].

For all dissolution experiments in the pure IL, a mixture of a metal oxide and [Hbet][NTf_2_] was heated to 175 °C for 24 h in a system open to the air to allow the evaporation of the arising water. Metal oxides were chosen in order to cover a wide range of different properties regarding the position of the metal in the periodic table, its oxidation state as well as the basicity of the oxide. An overview about their dissolution behaviour in [Hbet][NTf_2_] as well as [Hbet]_2_[NTf_2_]Cl is given in Table 1. The tested metal oxides included Al_2_O_3_, BaO, Bi_2_O_3_, CaO, Co_3_O_4_, Cr_2_O_3_, Cu_2_O, CuO, Fe_2_O_3_, Ga_2_O_3_, GeO_2_, In_2_O_3_, MgO, MnO, MnO_2_, MoO_3_, Nb_2_O_5_, NiO, PbO, PbO_2_, ReO_3_, Sb_2_O_3_, SnO, SrO, ThO_2_, TiO_2_, V_2_O_3_, V_2_O_5_, WO_3_ and ZnO. In order to provide enough ligands for complex formation, but at the same time minimise interfering signals of the unreacted IL, a typical metal ion-IL ratio of 1 : 4 was chosen. With this set-up, BaO, Bi_2_O_3_, CaO, Cu_2_O, CuO, MgO, MnO, PbO, PbO_2_, SrO, V_2_O_3_ and ZnO could successfully be dissolved in the IL, while Al_2_O_3_, Cr_2_O_3_, Fe_2_O_3_, Ga_2_O_3_, GeO_2_, In_2_O_3_, MnO_2_, MoO_3_, Nb_2_O_5_, NiO, ReO_3_, Sb_2_O_3_, ThO_2_, TiO_2_ and WO_3_ visually resulted in suspensions of the reagents. Only the liquid of the SnO sample was coloured brown, however, the ^1^H NMR spectrum shown in Fig. S9, ESI† reveals an intact IL. The colour of the liquid might result from very fine, dispersed SnO particles. An overview about the products obtained is given in Table S1, ESI.† In agreement with this, all reflections in the PXRD patterns of the latter samples could be attributed to the starting materials (see Fig. S4 and S5, ESI†). In contrast to this, the eight soluble metal oxides BaO, CaO, MgO, MnO, PbO, PbO_2_, SrO and ZnO gave homogenous solutions or pastes, whereby the high viscosity of the latter products could be lowered by using a higher amount of [Hbet][NTf_2_]. Apart from the relatively low-viscosity liquids of the CaO and MgO samples, crystallisation occurred during cooling or upon application on the PXRD sample holder, giving completely unidentified reflection patterns. Reflections at low 2*θ* angles suggest a large unit cell that might result from metal–betaine complex compounds.

**Table 1:** Experimentally observed solubility of metal oxides in water-free [Hbet][NTf_2_] and [Hbet]_2_[NTf_2_]Cl and their *U*/*x* values calculated from lattice energy data

Metal oxide	*U* [kJ mol^−1^]	*U*/*x* [kJ mol^−1^]	Solubility in [Hbet][NTf_2_]	Solubility in [Hbet]_2_[NTf_2_]Cl
Al_2_O_3_	15 464	7732	Negligible	Negligible
BaO	3121	3121	Full	Full (BaCl_2_ precipitation)
Bi_2_O_3_	13 318	6659	Low (in Ar flow)	Full (BiOCl precipitation)
CaO	3486	3486	Full	Full
Co_3_O_4_	18 067	6022	Low (reduction)	Full
Cr_2_O_3_	15 252	7626	Negligible	Negligible
Cu_2_O	3290	1645	High	Full (CuCl precipitation)
CuO	4150	4150	High	Full
Fe_2_O_3_	15 078	7539	Negligible	Low
Ga_2_O_3_	15 511	7756	Negligible	Negligible
GeO_2_	12 852	12 852	Negligible	Negligible
In_2_O_3_	14 439	7220	Negligible	Negligible
MgO	3889	3889	Full	Full
MnO	3798	3798	Full	Full
MnO_2_	13 075	13 075	Negligible	Full
MoO_3_	24 909	24 909	Low	Low
Nb_2_O_5_	34 030	17 015	Negligible	Negligible
NiO	4077	4077	Negligible	Low
PbO	3532	3532	Full	Full (PbCl_2_ precipitation)
PbO_2_	11 706	11 706	Full	Full (PbCl_4_ formation)
ReO_3_	24 433	24 433	Negligible	Negligible
Sb_2_O_3_	13 760	6880	Negligible	High
SnO	3662	3662	Negligible	Full
SrO	3322	3322	Full	Full
ThO_2_	9967	9967	Negligible	High
TiO_2_	12 114	12 114	Negligible	Negligible
V_2_O_3_	14 890	7445	Full	High
V_2_O_5_	38 738	19 369	Low (reduction)	Full
WO_3_	24 312	24 312	Negligible	Negligible
ZnO	4074	4074	Full	Full

**Fig. 2:**
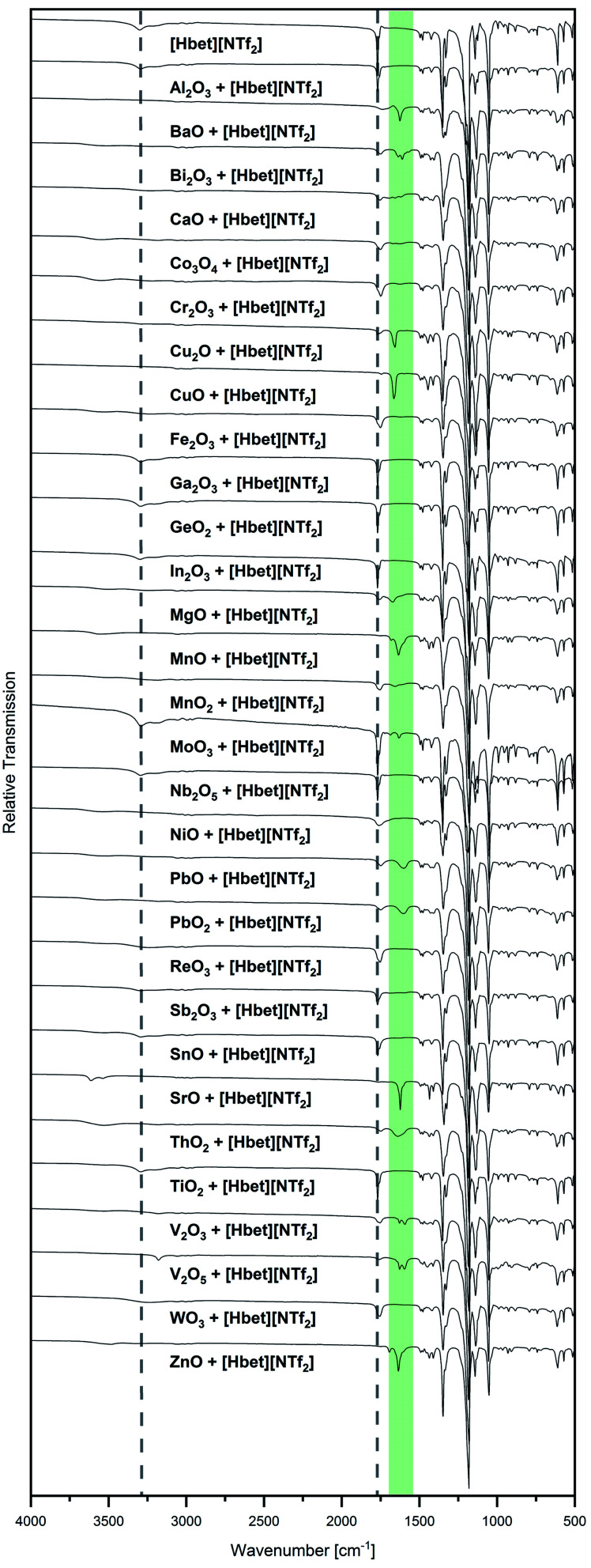
IR spectra of [Hbet][NTf_2_] and all samples of a metal oxide and [Hbet][NTf_2_] in the range of 500 cm^−1^ ≤ *ν̃* ≤ 4000 cm^−1^. The vibrational band positions of *ν*_as_ OH (3301 cm^−1^) and *ν*_as_ COO (1770 cm^−1^) of [Hbet][NTf_2_] are marked in dotted lines. The range of a shifted *ν*_as_ COO due to coordination of betaine is highlighted in green.

Besides visual examination and PXRD, evidence for complex formation is given by IR spectroscopy, where two bands attributed to the [Hbet]^+^ cation are a good indicator: the asymmetric stretching vibration of the carboxyl OH (*ν*_as_ OH) at 3301 cm^−1^ and the asymmetric stretching vibration of COO (*ν*_as_ COO) at 1770 cm^−1^ in the spectrum of the pure IL, which are marked with dotted lines in Fig. 2. Upon coordination, *ν*_as_ OH disappears due to the deprotonation of the carboxyl group. Meanwhile, a shift of *ν*_as_ COO to lower wavenumbers can be observed as the excitation of this vibration needs less energy when the carboxylate group binds to a heavier metal atom instead of a proton. In agreement with visual observations and PXRD patterns, no shift of *ν*_as_ COO is found for the samples of Al_2_O_3_, Co_3_O_4_, Cr_2_O_3_, Fe_2_O_3_, Ga_2_O_3_, GeO_2_, In_2_O_3_, MnO_2_, Nb_2_O_5_, NiO, ReO_3_, Sb_2_O_3_, SnO, TiO_2_ and WO_3_, indicating no significant dissolution of these metal oxides in [Hbet][NTf_2_]. As highlighted in green, a shift of this band as well as the disappearance of *ν*_as_ OH is observed for BaO, Bi_2_O_3_, CaO, Cu_2_O, CuO, MgO, MnO, PbO, PbO_2_, SrO, V_2_O_3_ and ZnO. The lack of correlation between the metal atom mass and the wavenumber of *ν*_as_ COO is attributed to different possible coordination modes of betaine, *i.e.* mono-, bidentate or bridging.

**Fig. 3:**
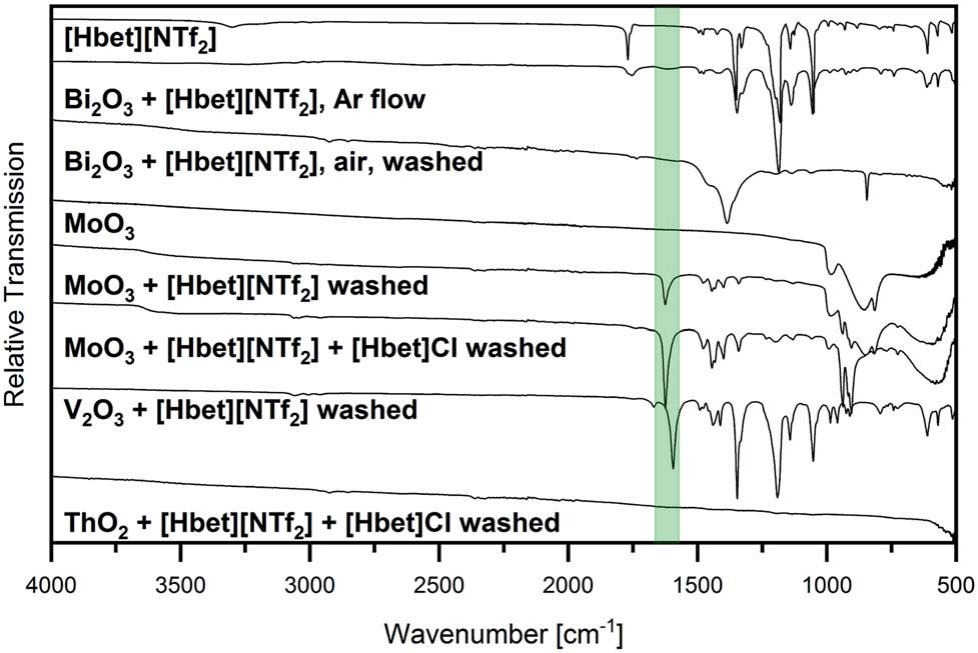
IR spectra of the sample Bi_2_O_3_ + [Hbet][NTf_2_] in argon flow as well as the samples of Bi_2_O_3_, MoO_3_, V_2_O_3_ + [Hbet][NTf_2_] and MoO_3_, ThO_2_ + [Hbet][NTf_2_] + [Hbet]Cl washed with acetone compared to the starting materials MoO_3_ and [Hbet][NTf_2_] in the range 500 cm^−1^ ≤ *ν̃*≤ 4000 cm^−1^. The position of *ν*_as_ COO is highlighted in green.

The IR spectra of several samples suggest the presence of metal–betaine complexes, however, precipitations occur. For CuO, no complete reaction was achieved, but small amounts of the starting material were present in a blue solution from which blue crystals precipitated. The same blue compound was obtained from Cu_2_O, as identified by PXRD, indicating the oxidation of copper(i). Similarly, the V_2_O_3_ sample contained a greenish blue powder in a colourless liquid. In order to clarify the nature of the solid, it was washed with acetone, hence, removing the IL. IR spectroscopy suggests the presence of V^3+^–betaine complexes due to the bands typical for betaine and [NTf_2_]^−^ but *ν*_as_ COO shifted to 1595 cm^−1^ (highlighted in green in Fig. 3). Furthermore, from Bi_2_O_3_, a white powder in a colourless liquid was obtained. After washing with acetone, it was identified as (BiO)_2_CO_3_ by PXRD (Fig. S5, ESI†). Bi_2_O_3_ is known to readily react with the acid anhydride CO_2_ to (BiO)_2_CO_3_,^18^ however, [Hbet][NTf_2_], apparently, significantly catalyses this reaction. In agreement with this, the IR spectrum in Fig. 3 is typical for this compound according to literature,^18^ indicating the absence of solid bismuth(iii)–betaine complex compounds. The repetition of the reaction under argon flow reveals a significantly lower reactivity of the reactants. Meeting general expectations, no reaction with CO_2_ takes place, but a suspension of a yellow powder in a colourless liquid forms. By PXRD, only reflections corresponding to the reagent Bi_2_O_3_ are found, which already contained some (BiO)_2_CO_3_ (Fig. S6, ESI†). However, the IR spectrum, displayed in Fig. 3, shows two *ν*_as_ COO bands at 1755 cm^−1^ and 1619 cm^−1^. This suggests a majority of unreacted IL alongside with a small amount of betaine coordinated to bismuth. Therefore, Bi_2_O_3_ is assumed to have a low solubility in [Hbet][NTf_2_]. The fast reaction on air might be ascribed to the precipitation of (BiO)_2_(CO_3_) shifting the reaction to the product side.

Some kind of special behaviour was also observed for Co_3_O_4_ and V_2_O_5_. Very small amounts of both oxides dissolve in the IL, yielding a violet, respectively green or sometimes brown liquid. As both metal oxides are strong oxidising agents,^2^ their partly dissolution is attributed to the oxidation of the IL. This is supported by the NMR spectra displayed in Fig S9,† where the occurrence of additional signals compared to pure [Hbet][NTf_2_] suggests the partial decomposition of the IL. In agreement with this, the PXRD pattern of the V_2_O_5_ sample product, shows several unidentified reflections besides the unreacted metal oxide. These might result from crystallised decomposition fragments of the IL. IR bands of low intensity at 1630 cm^−1^ and 1598 cm^−1^ are also attributed to the decomposition of the IL or small amounts of vanadium(iii)–betaine complexes. However, it also has to be taken into account, that the synthesis of [Hbet][NTf_2_] does not guarantee the complete absence of chloride. Therefore, also the oxidation of chloride impurities to chlorine by cobalt(iii) and vanadium(v) might play a role in the dissolution of the metal oxides.

Several low intensity IR bands in the range highlighted in green in Fig. 2 can also be found for the sample of MoO_3_, despite the unreacted appearance of the product as well as PXRD only identifying the reagents. IR spectroscopy of the remaining white powder washed with acetone gives evidence for the presence of molybdenum–betaine complexes, as shown in Fig. 3. The low intensity bands in the range 1300 cm^−1^ to 1500 cm^−1^ suggest the presence of betaine, while the nonappearance of the bands at lower wavenumbers might indicate the absence of [NTf_2_]^−^ anions. Furthermore, the occurrence of *ν*_as_ COO at 1626 cm^−1^, but no band at 1770 cm^−1^ gives evidence for the presence of betaine only in a coordinated state. Accordingly, MoO_3_ is suspected to show a low solubility in [Hbet][NTf_2_]. As a reason for this behaviour deviating from other MO_3_ type metal oxides, such as WO_3_ and ReO_3_, the crystal structure might serve. In contrast to the latter salts, MoO_3_ is built from layers of MoO_6_ octahedrons, which are expected to be affected by dissolution significantly easier than 3D cross-linked structures.

Similarly, the sample of ThO_2_ appears like a suspension of the reagents, but the IR spectrum shows a band at 1640 cm^−1^. However, PXRD of the starting material reveals some minor reflections besides the main pattern of ThO_2_, which could not be assigned to a phase and might result from the decay chain of ThO_2_ (Fig. S2, ESI†). Therefore, it is assumed that ThO_2_ does not dissolve in [Hbet][NTf_2_], but merely the impurities.

In order to substantiate these experimental observations, the dissolved, respectively undissolved metal oxides were investigated for common properties. A method to estimate the solubility of metal oxides in water-saturated [Hbet][NTf_2_] was recently suggested by Fan *et al.* who proposed a correlation between the lattice energy of a metal oxide and its solubility in the IL. Therefore, they introduced the *U*/*x* value of a metal oxide M_*x*_O_*y*_ where *U* is the lattice energy. For *U*/*x* < 7000 kJ mol^−1^, a good solubility of metal oxides was found while *U*/*x* > 10 000 kJ mol^−1^ indicated metal oxides insoluble in water-saturated [Hbet][NTf_2_]. Corresponding to their method,^9^ we calculated the lattice energies for all metal oxides examined experimentally by using the Born–Haber cycle. The resulting *U*/*x* values are listed in Table 1, detailed information about the data used for calculation can be found in Table S2, ESI.† In agreement with the assumptions of Fan *et al.*, the *U*/*x* value corresponds to the experimental results in most cases. Thus, also for systems of water-free [Hbet][NTf_2_], the dissolution of metal oxides can be expected for *U*/*x* < 7000 kJ mol^−1^. However, there are some exceptions from this general rule.

So no sign for dissolution is found for NiO despite its low lattice energy. Furthermore, Sb_2_O_3_ and V_2_O_3_ both possess *U*/*x* values around 7000 kJ mol^−1^, Co_3_O_4_ even lower with 6022 kJ mol^−1^, but only V_2_O_3_ with the highest *U*/*x* dissolves in [Hbet][NTf_2_]. Fan *et al.* also pointed out that there is no solubility for MO_2_ type metal oxides due to their typically high lattice energies. However, in our experiments, PbO_2_ formed a clear solution with the IL. Similarly, the partly dissolution of MoO_3_ is unexpected according to this rule, because of the high lattice energy. These deviations are in agreement with the report by Jayachandran *et al.* who claim that an increased temperature allows a significant dissolution of PuO_2_ in water-saturated [Hbet][NTf_2_],^19^ although predicted insoluble by Fan *et al.* with *U*/*x* = 10 381 kJ mol^−1^.^9^

From these observations it is concluded that the lattice energies of metal oxides, respectively their *U*/*x* values, merely allow a rough assessment of their solubility in [Hbet][NTf_2_]. The assumed reason is the lattice energy not being the only factor influencing solubility. Hence, the reaction conditions, such as temperature might have a significant influence, as assumed by Jayachandran *et al.*^19^ As illustrated by the example of MoO_3_ (layered structure) in contrast to WO_3_ and ReO_3_ (3D frameworks), the crystal structure of the starting materials might also have an effect on their disintegration. Furthermore, the product side of the reaction should not be neglected as complex formation constants as well as a possible change in the coordination number of the metal ion could affect the dissolution behaviour. A significant influence on the dissolution, in our opinion, also originates from the acidity or basicity of the metal oxide. Apparently, in the here performed experiments only relatively basic or amphoteric oxides readily react with the IL, meeting general expectations as [Hbet]^+^ is an acidic cation. However, these suggestions are based on qualitative observations that should be supported by measuring data. Yet, to our knowledge, no comprehensive table about the acid–base properties of metal oxides exists to provide such evidence.

### Dissolution of CuO and downstream chemistry

#### The compound [Cu_2_(bet)_4_(NTf_2_)_2_][NTf_2_]_2_

As a model system for downstream chemistry based on the dissolved metal oxide, CuO was chosen. The metal–betaine complex compound being present was identified by SCXRD as [Cu_2_(bet)_4_(NTf_2_)_2_][NTf_2_]_2_, crystallising in the monoclinic space group *P*2_1_/*n* (no. 14) with the lattice parameters *a* = 1445.50(4) pm, *b* = 1448.44(6) pm, *c* = 1498.92(4) pm and *β* = 95.182(3)° at 100 K.

**Fig. 4:**
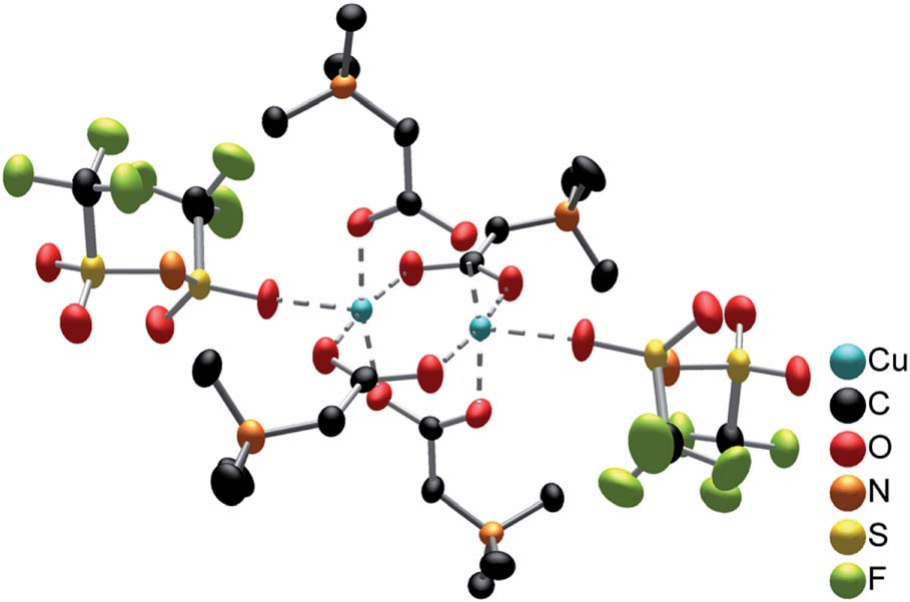
[Cu_2_(bet)_4_(NTf_2_)_2_]^2+^ cation. Cu^2+^ is shown in turquoise, C in black, O in red, N in orange, S in yellow and F in green. Coordinative interactions are marked as dotted lines. The ellipsoids enclose 70% of the probability density of the atoms at 100 K. H atoms are omitted for clarity.

The compound [Cu_2_(bet)_4_(NTf_2_)_2_][NTf_2_]_2_ consists of cationic [Cu_2_(bet)_4_(NTf_2_)_2_]^2+^ complexes and non-coordinating [NTf_2_]^−^ anions, as shown in Fig. S7, ESI.† The centroids of the complex cations (inversion centres) are located on the corners and in the centre of the unit cell. The dinuclear complex cation [Cu_2_(bet)_4_(NTf_2_)_2_]^2+^, displayed in Fig. 4, has a paddle-wheel structure with two Cu^2+^ ions coordinated by four μ-bridging betaine zwitterions *via* the carboxylate group. The distorted octahedral geometry is completed by Cu–Cu interactions (*d*(Cu–Cu) = 262.7 pm) and weakly O-coordinating [NTf_2_]^−^ anions at the axial positions. The coordinating oxygen atoms of betaine form an almost planar square around each copper(ii) ion in equal distance (194.5–196.5 pm). The *cis* angles between two adjacent betaine ligands (O_bet_–Cu–O_bet_) range from 88.6° to 90.6° while the *trans* angles between two opposing ligands amount to 168.8° indicating a considerable deviation from planarity. Perpendicular to this plane, a [NTf_2_]^−^ anion coordinates *via* an oxygen atom. The distance of 215.8 pm being 10% longer than the distance between copper(ii) and the coordinating oxygen atom of betaine, indicates weaker interactions.

The very similar complex cation [Cu_2_(bet)_4_(H_2_O)_2_]^2+^ was found in the compound [Cu_2_(bet)_4_(H_2_O)_2_][NTf_2_]_6_ by Nockemann *et al.* with two H_2_O molecules coordinating to copper(ii) instead of the [NTf_2_]^−^ anions.^4^ The distance between two copper(ii) ions in this compound (265.7 pm) is just slightly larger than in [Cu_2_(bet)_4_(NTf_2_)_2_]^2+^, while the distance between copper(ii) and the coordinating oxygen atom of betaine lies in the same range (195.8–197.3 pm). Weaker interactions are observed between copper(ii) and the coordinating aqua ligand (*d*(Cu–O_H_2_O_) = 213.3 pm).^4^ Also the structure-related complex [Cu_2_(betmMor)_4_(NTf_2_)_2_]^2+^ (betmMor = *N*-carboxy-methyl-*N*-methylmorpholinium) in the compound [Cu_2_(betMor)_4_][NTf_2_]_4_ represents a paddle-wheel complex with four betmMor ligands μ-bridging two copper(ii) ions and, perpendicular to this plane, two O-coordinating [NTf_2_]^−^ anions. The bridging betMor ligand is structurally related to betaine with two methyl groups on N replaced by a heterocyclic morpholine function. The interatomic distances (*d*(Cu–Cu) = 262.8 pm, *d*(Cu–O_betmMor_) = 196.1 pm and *d*(Cu–O_[NTf_2_]−_) = 218.3 pm)^5^ are in good agreement with the complex found in this work.

Further, similar [Cu_2_(bet)_4_]^4+^ paddle-wheel structures were reported, whereby the Cu^2+^ coordination sphere is completed by chloride and bromide anions instead of [NTf_2_]^−^. Examples are the complex [Cu_2_(bet)_4_Cl_2_]^2+^ in the compound [Cu_2_(bet)_4_Cl_2_]Cl_2_·4H_2_O^20^ and [Cu_2_(bet)_4_Br_2_]^2+^ in the compounds [Cu_2_(bet)_4_Br_2_]Br_2_·2H_2_O^21^ and [Cu_2_(bet)_4_Br_2_][CuBr_4_]·H_2_O.^22^ The coordination of the strong chloride and bromide ligands results in a relatively large distance between the two copper(ii) ions (276.6 pm, 276.8 pm, 278.3 pm, respectively).^20–22^ This supports the assumption of weaker interactions between copper(ii) and the [NTf_2_]^−^ ligand in [Cu_2_(bet)_4_(NTf_2_)_2_]^2+^ with a significantly shorter *d*(Cu–Cu).

Many further examples of copper paddle-wheel structures showing the same characteristics, such as the Cu–Cu distance, the distance ratio of coordination bonds and a distorted octahedral geometry, are known in literature.^23,24^

A comparison of the experimental PXRD pattern of the CuO–[Hbet][NTf_2_] reaction product to the one simulated from single-crystal data of [Cu_2_(bet)_4_(NTf_2_)_2_][NTf_2_]_2_ gives evidence for the phase impurity of the synthesis product, as shown in Fig. S8, ESI.† All theoretical reflections turn up in the experimental pattern, whereby the shift to lower 2*θ* values meets the expectations due to the temperature difference of PXRD (RT) and SCXRD (100 K) measurements. By Rietveld refinement, a room temperature unit cell was obtained with 0.9%, 1.5% and 1.6% enlarged *a*, *b* and *c* lattice parameters, respectively (*a* = 1459.0 pm, *b* = 1470.2 pm, *c* = 1522.9 pm, *β* = 95.3°).

Besides, apparently, another unidentified phase is present, as numerous reflections cannot be ascribed to any known phase. Attempts to determine a possible unit cell were not successful, while a microscopic separation by crystal morphology also is not possible due to irregular crystal shapes. However, due to the low 2*θ* angles of several reflections, indicating a similarly large unit cell, another unknown copper–betaine compound phase should be considered.

#### Ligand exchange experiments

In order to investigate whether the weakly coordinating [NTf_2_]^−^ anions can be replaced by other ligands, the reaction of [Cu_2_(bet)_4_(NTf_2_)_2_][NTf_2_]_2_ in [P_66614_]Cl was investigated. In agreement with visual examinations, IR and Raman spectroscopy, an exchange of all ligands by chloride and, hence, the formation of [CuCl_4_]^2−^ anions is assumed.

**Fig. 5:**
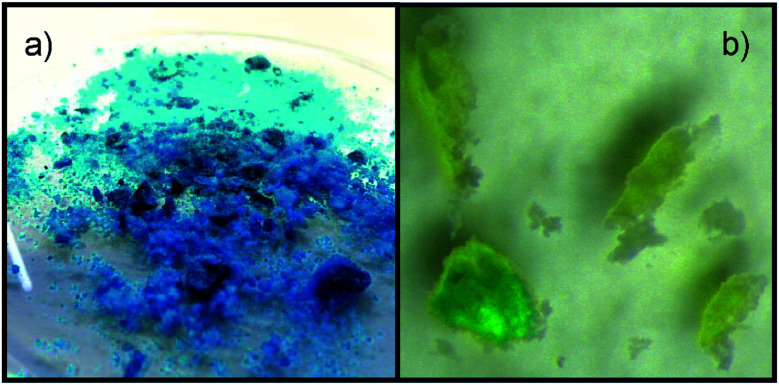
Images of (a) [Cu_2_(bet)_4_(NTf_2_)_2_][NTf_2_]_2_ and (b) the discolouration in [P_66614_]Cl accompanied by the yellowing of the surrounding IL.

Stirring a mixture of the blue complex compound and [P_66614_]Cl, an immediate yellow colouring of the liquid as well as an discolouration of the blue powder after several hours is observed. For a closer investigation of the processes taking place, a crystal of [Cu_2_(bet)_4_(NTf_2_)_2_][NTf_2_]_2_ was covered with one drop of [P_66614_]Cl, revealing a discolouration of the crystal beginning from the surface accompanied by the yellow colouring of the IL in the surroundings, as shown in Fig. 5. The remaining colourless crystal was identified as [Hbet]Cl by SCXRD. It crystallises in the monoclinic space group *P*2_1_/*c* with the cell parameters *a* = 743.6 pm, *b* = 898.4 pm, *c* = 1150.0 pm and *β* = 97.0° at 100 K, in agreement with literature.^25,26^

**Fig. 6:**
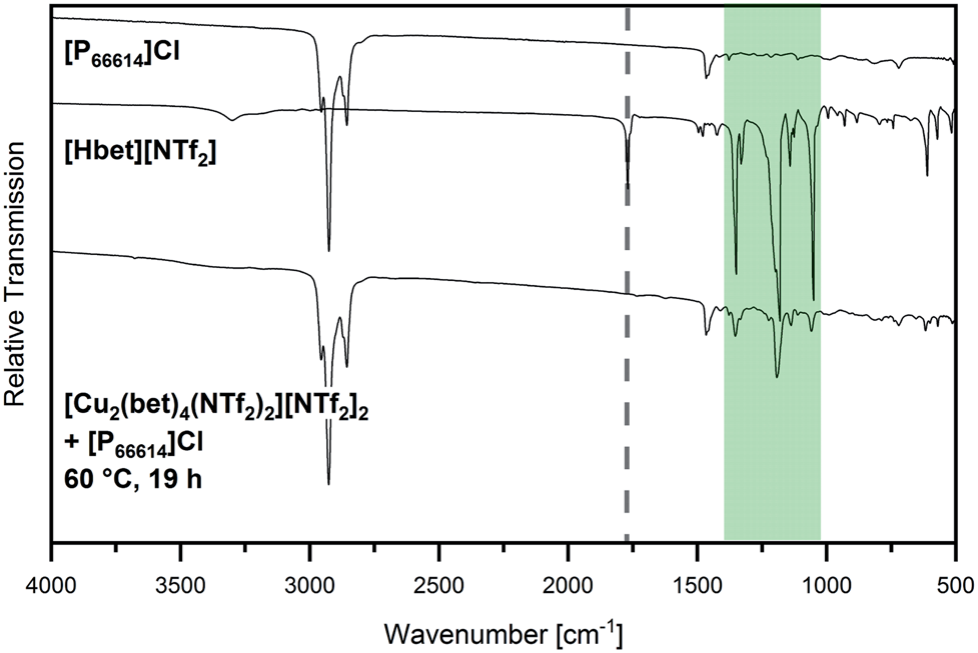
IR spectrum of [Cu_2_(bet)_4_(NTf_2_)_2_][NTf_2_]_2_ that was stirred at 60 °C in [P_66614_]Cl for 19 h (bottom) compared to the spectra of [Hbet][NTf_2_] (middle) and [P_66614_]Cl (top) in the range 500 cm^−1^ ≤ *ν̃* ≤ 4000 cm^−1^. The position of *ν*_as_ COO of [Hbet]^+^ is marked with a dotted line and the region of the most intensive bands of [Hbet][NTf_2_] is highlighted in green.

The consequent conclusion of [Hbet]^+^ cations not being dissolved in [P_66614_]Cl is supported by IR spectroscopy, as shown in Fig. 6. Clearly observable are the main peaks of [P_66614_]Cl, but also the most intensive signals of [Hbet][NTf_2_] are visible in the spectrum of the sample (highlighted in green). According to literature^27–30^ all of them are attributed to vibrational modes of the [NTf_2_]^−^ anion (see Table S3, ESI†), while *ν*_as_ COO at 1770 cm^−1^ (respectively the shifted signal of coordinated [Hbet]^+^ at 1655 cm^−1^) is not observed despite its medium intensity.

**Fig. 7:**
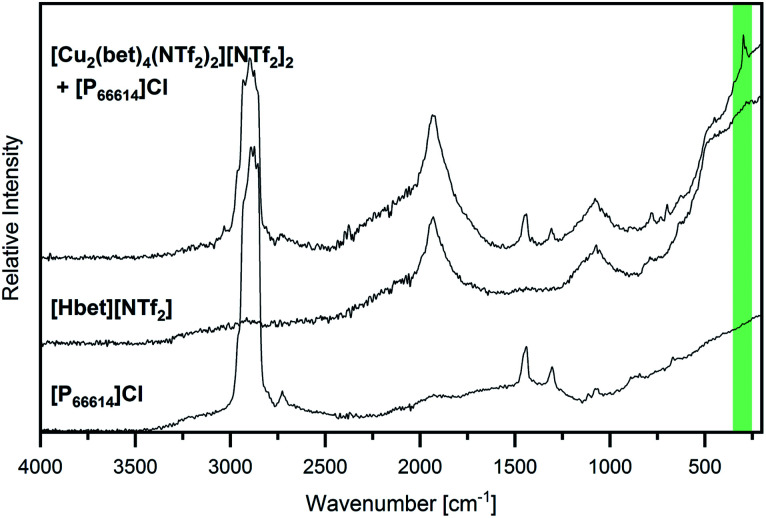
Raman spectrum of [Cu_2_(bet)_4_(NTf_2_)_2_][NTf_2_]_2_ that was stirred at 60 °C in [P_66614_]Cl for 19 h (top) compared to the spectra of [Hbet][NTf_2_] (middle) and [P_66614_]Cl (bottom) in the range 200 cm^−1^ ≤ *ν̃* ≤ 4000 cm^−1^. The position of *ν*_s_ CuCl is highlighted in green.

Further evidence of copper(ii) being leached from the [Cu_2_(bet)_4_(NTf_2_)_2_]^2+^ complex is given by the Raman spectrum displayed in Fig. 7. A band at 297 cm^−1^ suggests a Cu–Cl stretching vibration (*ν*_s_ CuCl, highlighted in green),^31,32^ while no signal is found around 385 cm^−1^, where Cu–O vibrations would be expected.^31^ Furthermore, Suffren *et al.* reported a correlation of the band position of *ν*_s_ CuCl and the *trans* angles in [CuCl_4_]^2−^ complex anions, whereby they found a shift to higher wavenumbers with increasing tetrahedral distortion over a square planar geometry.^33^ Therefore, the experimental Raman band at a relatively high wavenumber is in good agreement with the yellow colour of the solution suggesting a distorted tetrahedral geometry for [CuCl_4_]^2−^. A distorted tetrahedron is the high-temperature phase of the thermochromic [CuCl_4_]^2−^. Despite being the electrostatically more favoured geometry, it is assumed that the square-planar RT phase is stabilized by hydrogen bonds at lower temperatures.^34^ As in an environment of [P_66614_]^+^ cations, hydrogen bonds to [CuCl_4_]^2−^ are not likely, the distorted tetrahedral geometry of this complex anion seems reasonable.

Altogether, these investigations suggest that copper(ii) ions are leached from the Cu–betaine complex and form [CuCl_4_]^2−^ complex anions in a distorted tetrahedral geometry. Thus, a complete exchange of the O-coordination sphere around copper(ii) by chloride could be shown, opening promising possibilities for downstream chemistry starting from metal oxides.

### The role of chloride for the dissolution of metal oxides

**Fig. 8:**
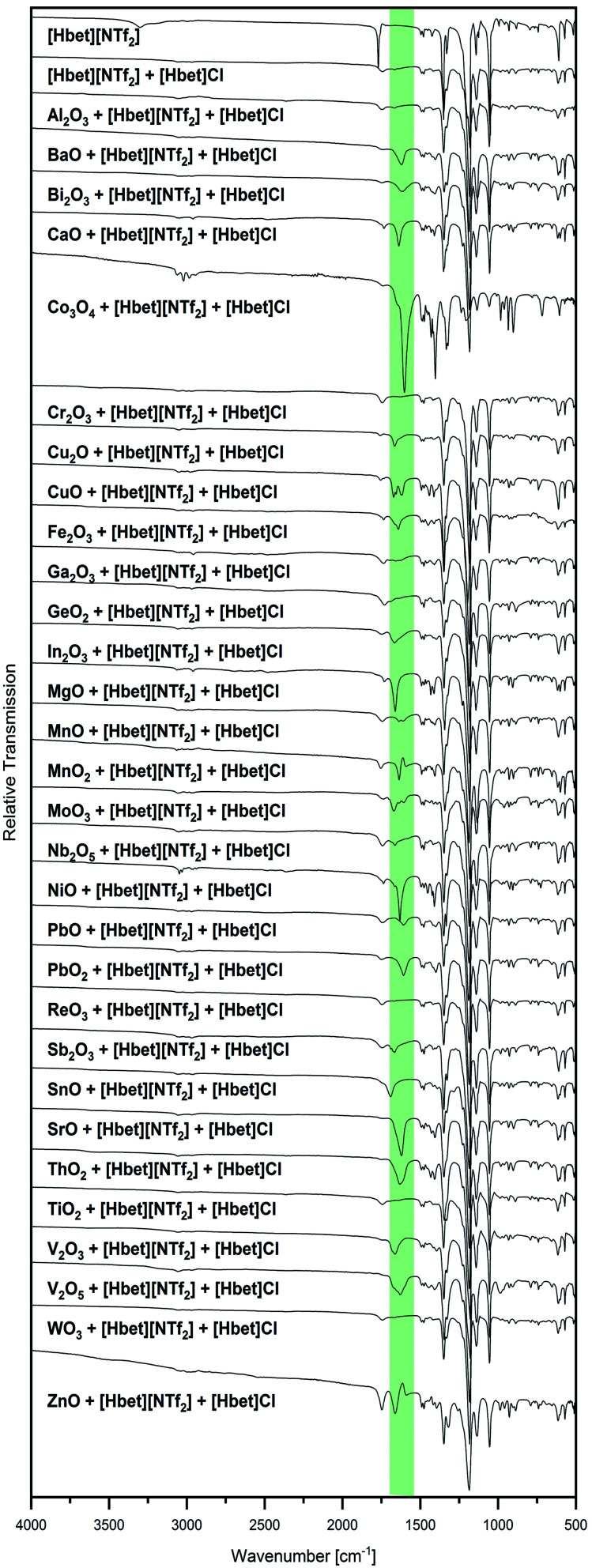
IR spectra of [Hbet][NTf_2_], [Hbet]_2_[NTf_2_]Cl and all samples of a metal oxide in [Hbet]_2_[NTf_2_]Cl stirred at 175 °C for 24 h in the range of 500 cm^−1^ ≤ *ν̃* ≤ 4000 cm^−1^. The range of a bands attributed to *ν*_as_ COO of a coordinated betaine carboxylate group is highlighted in green.

The role of a strong nucleophile as potential additional ligand in the dissolution of metal oxides was investigated by the addition of [Hbet]Cl, thus introducing chloride anions without any other, possibly interfering cations. Typically, a 1 : 1 mixture of [Hbet][NTf_2_] and [Hbet]Cl was used. Samples with the ratio of metal cations to [Hbet]_2_[NTf_2_]Cl of 1 : 2 were investigated under the same conditions like in the previous experiments. However, ^1^H NMR spectroscopy gives evidence for the decomposition of the IL by a significant increase in the number of signals observed. In the spectrum of [Hbet][NTf_2_] displayed in Fig. S9, ESI,† the peaks at 3.1 ppm and 4.1 ppm are attributed to the protons of the CH_3_ and CH_2_ groups of betaine, respectively. Another signal of the carboxyl proton is not observed which is attributed to its low intensity and the expected broadness.^35^ Due to the complexity of the spectrum obtained from heated [Hbet]_2_[NTf_2_]Cl, no attribution to specific decomposition fragments was realised, yet. Concomitant in the IR spectrum of the resulting brown liquid (Fig. 8), the sharp, medium-intensity *ν*_as_ COO band at 1770 cm^−1^ transforms into two low intensity bands at 1743 cm^−1^ and 1666 cm^−1^. The first band is attributed to *ν*_as_ COO of undecomposed [Hbet]^+^, while the second band is assumed to result from a not yet identified decomposition fragment. In Fig. S9, ESI,†^1^H NMR spectra of several samples give evidence for the decomposition of the IL taking place in mixtures with metal oxides as well. Further studies of several systems indicate, that this can be reduced by increasing the amount of [Hbet][NTf_2_] as well as by decreasing the reaction time.

The influence of chloride anions on the dissolution of a metal oxide was investigated in detail on the example of CuO. As a result of this, a general dissolution promoting effect of chloride can be stated, however, the specific impact strongly depends on the amount of [Hbet]Cl added.

On one side, the addition of chloride in small amounts (0.3 mol% based on the total number of counter ions of [Hbet]^+^) reveals a strong catalytic effect. Thus, in a reaction for 24 h, the yield of [Cu_2_(bet)_4_(NTf_2_)_2_][NTf_2_]_2_ could be increased from 13% to 66% by the addition of [Hbet]Cl. Such a promoting effect of chloride anions is not uncommon, *e.g.* was a similar outcome observed for the synthesis of Cu_3−*x*_P from copper and red phosphorous, whereby the product yield could significantly be increased by the addition of a few mol% of [P_66614_]Cl to [P_66614_][NTf_2_].^36^

**Fig. 9:**
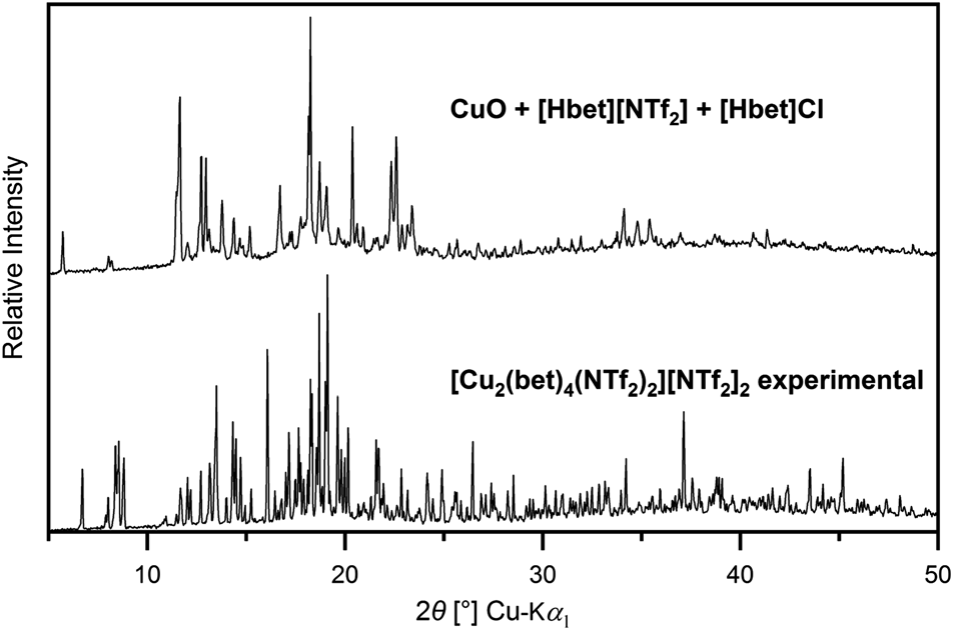
Experimental diffractogram of the sample CuO + [Hbet][NTf_2_] + [Hbet]Cl (4 h reaction time, top) compared to the experimental diffractogram of [Cu_2_(bet)_4_(NTf_2_)_2_][NTf_2_]_2_ in the range 5° ≤ 2*θ* ≤ 90°.

While the ideal reaction time for the sample with 0.3 mol% chloride amounts to 24 h, by the addition of equimolar amounts of [Hbet]Cl, *i.e.* [Hbet]_2_[NTf_2_]Cl, it reduces to only 4 h. However, besides an effect on the reaction time, also an influence on the product phase is observed. As shown in Fig. 9, the PXRD pattern does not correspond to the pattern of [Cu_2_(bet)_4_(NTf_2_)_2_][NTf_2_]_2_. Instead, the low 2*θ* angles of the numerous unidentified reflections suggest a large unit cell that might result from another copper–betaine complex compound. Evidence for this is found in the IR spectrum displayed in Fig. 8. A low intensity band at 1753 cm^−1^ might result from a small amount of uncoordinated [Hbet]^+^, while another band at 1669 cm^−1^ might be conform with the respective band in the [Cu_2_(bet)_4_(NTf_2_)_2_][NTf_2_]_2_ spectrum resulting from bridging coordination of betaine. However, two additional bands at 1655 cm^−1^ and 1618 cm^−1^ are solely found in the spectrum of the chloride-rich sample and might indicate another coordination of betaine to copper(ii), *i.e.* monodentate or bidentate. Thus, the positive effect of chloride anions on the reaction rate, as suggested by the group of Binnemans,^8^ can be confirmed. Hence, this property is not unique to water, but H_2_O in principle appears to be substitutable by other small molecules or ions.

**Fig. 10:**
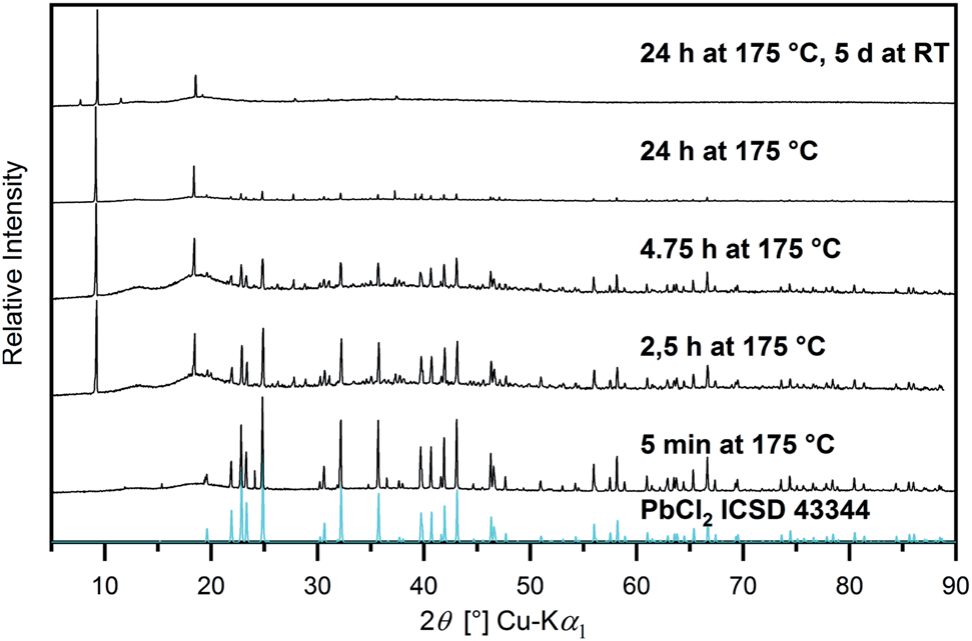
PXRD patterns of the sample PbO + [Hbet][NTf_2_] + [Hbet]Cl (*n*_Pb_ : *n*_[Hbet][NTf_2_]_ : *n*_[Hbet]Cl_ = 1 : 4 : 2) after different reaction times compared to the diffractogram of PbCl_2_ simulated from single crystal data (blue) in the range 5° ≤ 2*θ* ≤ 90°.

In order to investigate whether chloride ions are only suitable to decrease reaction time or might also support the dissolution of so far insoluble metal oxides, their effect in other reaction systems was examined. In agreement with the previous findings, significantly more metal oxides compared to the pure IL could be dissolved by the addition of chloride. Thus, in a 1 : 1 mixture of [Hbet][NTf_2_] and [Hbet]Cl, Co_3_O_4_, Fe_2_O_3_, MnO_2_, NiO, Sb_2_O_3_, SnO, ThO_2_, and V_2_O_5_ apparently form metal–betaine complexes to some extent, which does not occur in the pure IL. Additionally, also BaO, Bi_2_O_3_, CaO, Cu_2_O, CuO, MgO, MnO, MoO_3_, PbO, PbO_2_, SrO, V_2_O_3_ and ZnO appear to react with [Hbet][NTf_2_] in the presence of chloride, as well. Information about the products obtained are given in Table S4, ESI.† While Sb_2_O_3_, SrO and ZnO give clear solutions with [Hbet][NTf_2_] in the presence of [Hbet]Cl, for the CaO and MgO samples, solid products were obtained and for MnO, a paste. By further dilution with [Hbet][NTf_2_], the three metal oxides dissolved and yielded clear solutions as well. In contrast to this, for the samples of BaO and Cu_2_O, the powders present in the product suspensions were identified as the respective chloride salts by PXRD, while for Bi_2_O_3_, BiOCl was found. Apparently, the interactions of the metal ions with chloride are favoured over betaine, leading to precipitation following dissolution of the oxide. The reaction to chloride salts is, furthermore, found for the two lead oxides, however, they appear to be unstable as time-resolved PXRD measurements reveal (Fig. 10). Thus, for the sample of PbO a white precipitation occurs immediately after the melting of the IL and, after 5 minutes reaction time, PbCl_2_ is identified as the only crystalline phase by PXRD. With increasing time, the PbCl_2_ phase disappears, giving way for another, yet unidentified phase. This change of composition continues during storage of the sample at room temperature. It is conceivable that a partly ligand exchange reaction takes place, yielding Pb–Cl–betaine complexes or the decomposition products of the IL (Fig. S9, ESI†) might play a role in these observations. Similarly, for the sample of PbO_2_, a yellow colouring of the molten IL takes place, suggesting the presence of PbCl_4_. According to the literature,^2^ this metastable liquid decomposes into solid PbCl_2_ and Cl_2_ gas above 50 °C. However, PbCl_2_ could not be found by PXRD, which is attributed to further reactions according to the PbO sample. Several experiments in the PbO and PbO_2_ systems showed that a relatively large variety of diffraction patterns with unidentified phases is obtained, whereby no rational rule could be determined as causal, yet. The numerous different phases occurring during the reactions should be investigated by further experiments.

Despite the precipitation of chloride salts in several cases, the IR spectra display a *ν*_as_ COO band shifted to lower wavenumbers towards the pure IL (highlighted in green in Fig. 8). This suggest the presence of some amount of metal ions coordinated by betaine for these samples. The same assumption is made for MnO_2_, where an unidentified solid precipitation was obtained in terms of numerous colourless crystals in a brown liquid. The crystal colour clearly deviating from the black reagent suggests dissolution.

Furthermore, for the mentioned metal oxides, assumed dissolved, the reagent oxide cannot be identified by PXRD, as shown in Fig. S10 and S11, ESI.† This is different from Fe_2_O_3_, NiO, V_2_O_3_ and ThO_2_. From Fe_2_O_3_ a red powder identified as Fe_2_O_3_ by PXRD was obtained in a brown liquid. Additional unidentified reflections are attributed to decomposition fragments of the IL in agreement with the ^1^H NMR spectrum in Fig. S9, ESI.† However, two bands in the IR spectrum at 1737 cm^−1^ and 1641 cm^−1^ suggest the occurrence of uncoordinated as well as coordinated betaine. The presence of dissolved iron(iii) in the separated liquid phase was confirmed by testing with aqueous KSCN and NaF solutions. Therefore, a low dissolution for Fe_2_O_3_ is assumed. For the solid NiO sample product, a medium intensity IR band at 1632 cm^−1^ suggests the coordination of betaine. Further evidence for the partly dissolution of NiO is given by the addition of acetone, which according to our experience is a suitable solvent for some metal–betaine complexes, but not for the respective oxides. Thus, a green liquid is obtained, indicating the presence of dissolved nickel(ii) ions, alongside with green NiO powder. Similarly, for V_2_O_3_, a suspension of a brown paste and a black powder is obtained, which is identified as V_2_O_3_ by PXRD. Changing the reagent ratio to *n*_V_ : *n*_[Hbet][NTf_2_]_ : *n*_[Hbet]Cl_ = 1 : 6 : 1 yields a clear solution with only a few black particles. Corresponding to this, the IR spectrum shows a band at 1662 cm^−1^ suggesting the coordination of betaine to vanadium(iii).

In contrast to this, ThO_2_ appears to react to a white, amorphous powder. Only ThO_2_ is identified by PXRD, which is attributed to a small amount of black particles in the sample. Hence, no complete reaction was achieved (neither with triple the amount of IL). However, EDX gives evidence for the white powder being an organometallic thorium compound with Th, O and C in an approximate ratio of 2 : 7 : 10 (Fig. S12 and Table S5, ESI†). As only semiquantitative measurement conditions were applied and H atoms cannot be detected by EDX, no assumptions of the precise composition of this compound should be made. However, as no N is detected, the presence of thorium–betaine complexes appears unlikely. Instead, the powder might be formed by thorium and a decomposition product of the IL. This is in agreement with the IR spectra of the powder washed with acetone, as shown in Fig. 3. No bands are observed for the washed powder indicating the absence of betaine as well as [NTf_2_]^−^ ions. Further experiments in this system should be performed in order to investigate whether the precipitation of a thorium compound can be avoided by suppressing the decomposition of the IL and to clarify the nature of this white powder.

Similar to the reaction in a chloride-free system, also the dissolution of very small amounts of MoO_3_ is suspected due to several bands in the highlighted range in the IR spectrum. As PXRD indicates the presence of unreacted MoO_3_, the product was washed with acetone and again studied by IR spectroscopy, giving evidence for molybdenum–betaine complexes, as shown in Fig. 3. Hence, a low solubility of MoO_3_ is assumed in the presence of chloride as well as in the pure IL.

For other products, such as Al_2_O_3_, Ga_2_O_3_, GeO_2_, Nb_2_O_5_, ReO_3_, TiO_2_ and WO_3_, the reagent metal oxide is present in a brown liquid and no dissolution is assumed. The respective IR spectra typically correspond to uncoordinated betaine in a [Hbet][NTf_2_]–[Hbet]Cl mixture and PXRD patterns show unidentified reflections. This is attributed to decomposition fragments of the IL crystallising. For the presumably undissolved metal oxide In_2_O_3_, the two bands in the IR spectrum at 1743 cm^−1^ and 1666 cm^−1^ are present in intensity proportions deviating from metal-free [Hbet]_2_[NTf_2_]Cl. This is attributed to varied amounts of decomposition fragments of the IL, but as the band positions are similar, no complex formation is assumed. In contrast to this, in the Cr_2_O_3_ sample, the metal oxide is present in a colourless liquid. However, the ^1^H NMR spectrum in Fig. S9, ESI† reveals the partly decomposition of the IL. The IR spectrum does not suggest any dissolution.

Altogether, again, a general trend is observed, that very acidic metal oxides do not react with [Hbet]^+^, but only basic or amphoteric ones. However, as V_2_O_5_ and Co_3_O_4_ form an exception from this trend, another dissolution mechanism was identified as causal. Although the black powder in the V_2_O_5_ sample could not be identified by PXRD, it clearly is not the yellow reagent V_2_O_5_. Instead, a green colour in solution usually is associated with vanadium(iii),^2^ suggesting a redox reaction. The IR spectra shows only one broad band at 1628 cm^−1^ that might result from betaine coordinated to vanadium(iii). As V_2_O_5_ is known to oxidize chloride to chlorine,^2^ the reduction of vanadium(v) to vanadium(iii) by the oxidation of chloride is conceivable. However, the ^1^H NMR spectrum in Fig. S9, ESI† also suggests the decomposition of the IL.

Similarly, the dissolution of Co_3_O_4_ is attributed to a redox reaction. Many small, blue crystals were obtained as reaction product. As CoCl_2_ could be ruled out by a different PXRD pattern and differing physical properties (less hygroscopic, different dissolution behaviour in acetone), the small crystals are assumed to consist of cobalt–betaine complexes. The blue colour, respectively the violet colour of an aqueous solution, suggest an oxidation state of cobalt(ii), as cobalt(iii) would be expected to form green aqua-complexes.^37^ This is in agreement with the known oxidising effect of cobalt(iii) in the presence of chloride by the formation of chlorine,^2^ but the ^1^H NMR spectrum also suggests the decomposition of the IL. Therefore, the nature of the reductant of V_2_O_5_ and Co_3_O_4_ is uncertain, yet.

Summarising, for the here studied metal oxides, the presence of chloride in several cases has a positive effect on the dissolution. So in a 1 : 1 mixture of [Hbet][NTf_2_] and [Hbet]Cl, Co_3_O_4_, Fe_2_O_3_, MnO_2_, NiO, Sb_2_O_3_, SnO, ThO_2_, and V_2_O_5_ apparently form metal–betaine complexes, which does not occur in the pure IL. Furthermore, complex formation is assumed for BaO, Bi_2_O_3_, CaO, Cu_2_O, CuO, MgO, MnO, MoO_3_, PbO, PbO_2_, SrO, V_2_O_3_ and ZnO in the pure IL as well as in the presence of chloride. Altogether, the addition of chloride ions enables not only the dissolution of more metal oxides than in pure [Hbet][NTf_2_], but, as studied in detail for the CuO, system, also has a decreasing effect on the reaction time and might affect the product phase. This reaction-promoting effect of small co-ligands is in agreement with numerous investigations of other research groups.^4,7,8,38^ However, not only water molecules can be such co-ligands, but the extensive investigations in this work give evidence for the applicability of chloride ions. Nevertheless, it has to be considered, that several metal ions form not readily soluble chloride salts after dissolution, which might complicate downstream chemistry. Altogether, the dissolution of metal oxides in task-specific reaction systems seems possible. Further investigations have to be performed to determine whether the chloride ions act as permanent ligands in metal–betaine complexes or exhibit a catalytic effect. Furthermore, experiments with different small ions or molecules as potential co-ligands will be interesting.

## Conclusions

[Hbet][NTf_2_] is a suitable IL for the dissolution of various, basic or amphoteric metal oxides by the formation of metal–betaine complexes. The dissolution is assumed to be influenced by a combination of various factors, whereas the basicity is considered important, but also the lattice energy of the metal oxide, the crystal structure and the reaction temperature play a role. Furthermore, a decrease of the reaction rate in the absence of water can be confirmed. The addition of chloride ions improves the solubility of metal oxides. Thereby, the amount of chloride in the reaction mixture appears to not only have an effect on the reaction time by means of a catalyst, but also to affect the product phase.

Closer investigations of the reaction of CuO in [Hbet][NTf_2_] revealed the new complex compound [Cu_2_(bet)_4_(NTf_2_)_2_][NTf_2_]_2_. Regarding downstream chemistry, it was shown that by addition of this compound to the IL [P_66614_]Cl a ligand exchange reaction takes place, yielding [CuCl_4_]^2−^ anions. Thus, a proof of concept for the dissolution of a metal oxide in [Hbet][NTf_2_] with a subsequent complete replacement of the O-coordination sphere by other ligands was provided. This opens up a new, low-temperature approach for the transformation of metal oxides into chemicals in demand, that further research might make applicable to naturally occurring ores, earths and minerals.

## Conflicts of interest

There are no conflicts to declare.

## Supplementary Material

RA-009-C9RA06423K-s001

RA-009-C9RA06423K-s002
