# Supplementary material for: Dissolution of metal oxides in task-specific ionic liquid
Source: RSC Adv. 2019 Sep 19;9(51):29699–710. doi: 10.1039/c9ra06423k (PMC9071953; doi:10.1039/c9ra06423k)
Supplement: RA-009-C9RA06423K-s001 [file RA-009-C9RA06423K-s001.pdf]

Electronic Supplementary Material (ESI) for RSC Advances.
This journal is © The Royal Society of Chemistry 2019

Electronic Supporting Information

Dissolution of metal oxides in task-specific ionic liquid

Janine Richter,[a] Michael Ruck[a,b]

[a] Technische Universität Dresden

[b] Max-Planck-Institute for Chemical Physics of Solids, Dresden

PXRD of the reagents ThO_2 and [Hbet][NTf_2]

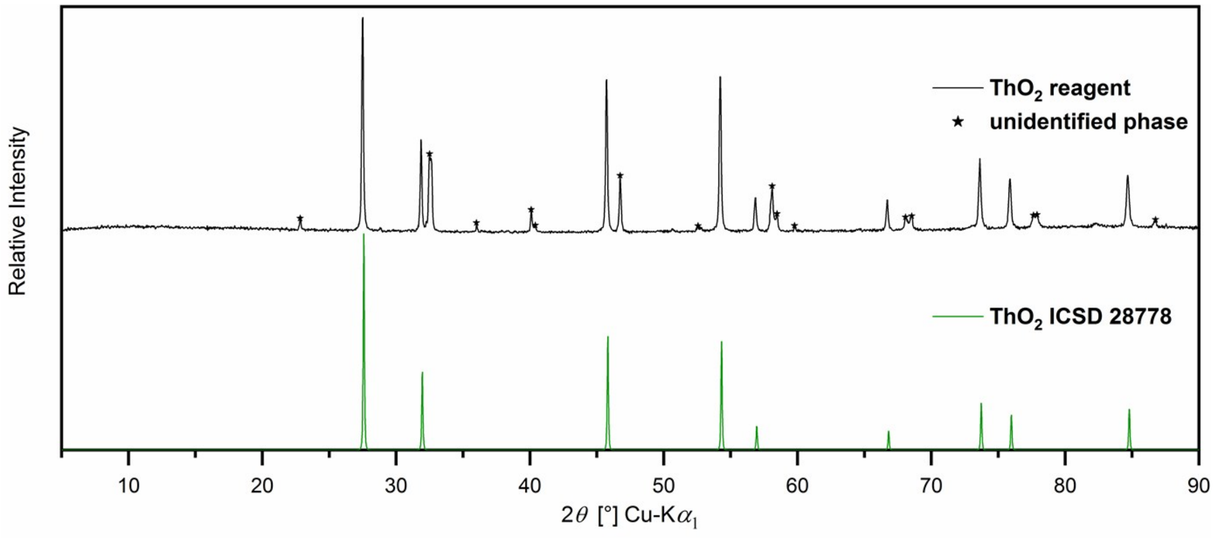

Fig. S1 Measured diffractogram of the reagent ThO_2 (black) compared to the ThO_2 pattern calculated from single-crystal data (green) in the range $5° \leq 2\theta \leq 90°$.

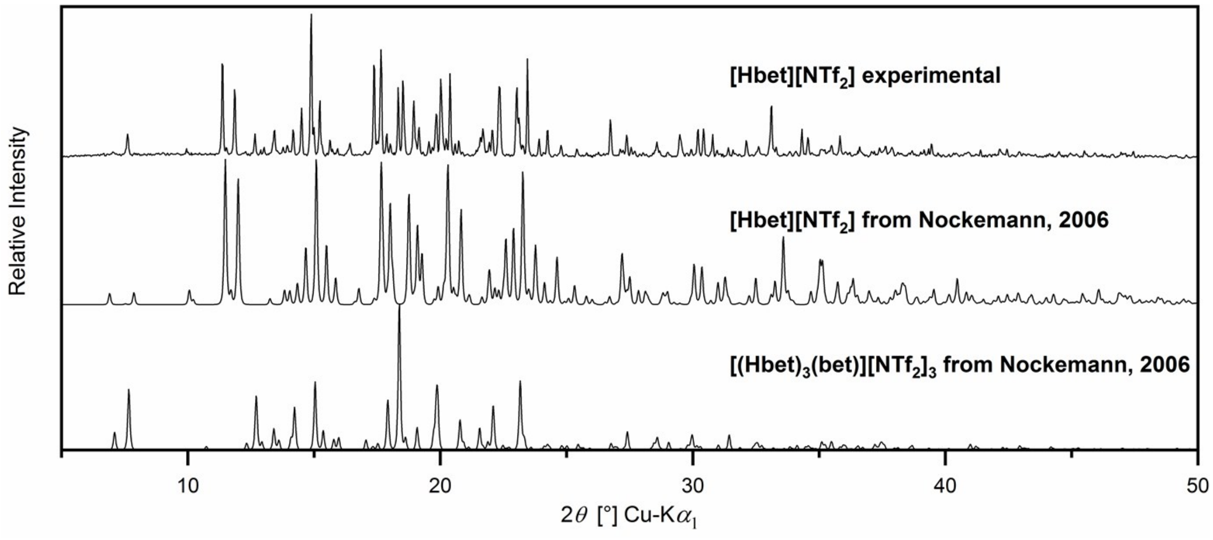

Fig. S2 Experimental diffractogram of the synthesised [Hbet][NTf_2] compared to the reflection patterns of [Hbet][NTf_2] (middle) and [$(Hbet)_3$(bet)][NTf_2]$_3$ (bottom) simulated from single crystal data[1] in the range $5° \leq 2\theta \leq 50°$.

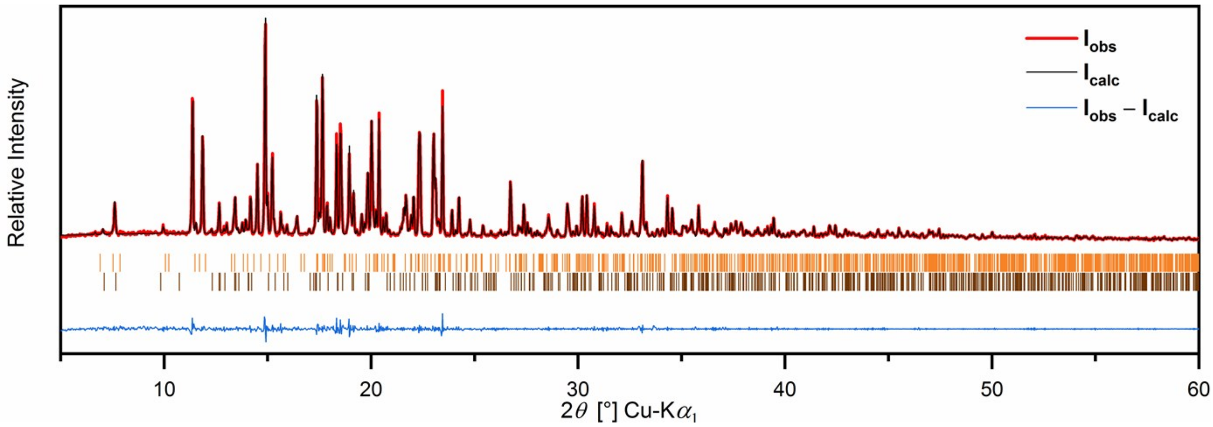

Fig S3 Rietveld refinement plot of the reagent [Hbet][NTf_2]. Peak positions of [Hbet][NTf_2] are marked in orange and of [$(Hbet)_3$(bet)][NTf_2]$_3$ in brown vertical bars. Ca. 66 % of [Hbet][NTf_2] and 34 % of [$(Hbet)_3$(bet)][NTf_2]$_3$ are present in the sample. $R_p = 3.8102$, $R_{wp} = 5.3084$, $R_{exp} = 7.7653$, GOF = 0.68.

Reaction mixtures in pure [Hbet][NTf_2]

Table S1 Product appearance and phases identified by PXRD of the reaction mixtures of a metal oxide and [Hbet][NTf_2]. A molar ratio of $n_M : n_{IL} = 1 : 4$ and heating to 175 °C for 24 h was applied. Paste-like products crystallised when brought on a PXRD sample holder.

Oxide	Product appearance	PXRD phases
Al_2O_3	White powder in colourless liquid	[Hbet][NTf_2]
BaO	White paste	Many unidentified reflections
Bi_2O_3	White powder in colourless liquid	Many unidentified reflections
CaO	Clear, colourless solution	-
Co_3O_4	Black powder in slightly violet liquid	Co_3O_4
Cr_2O_3	Green powder in colourless liquid	Cr_2O_3
Cu_2O	Blue and white crystals	Cu_2O, [Hbet][NTf_2], [$Cu_2(bet)_4(NTf_2)_2$][NTf_2]$_2$
CuO	Blue and white irregular crystals	[$Cu_2(bet)_4(NTf_2)_2$][NTf_2]$_2$, unidentified reflections
Fe_2O_3	Red powder in colourless liquid	Fe_2O_3
Ga_2O_3	White powder in colourless liquid	Ga_2O_3, [Hbet][NTf_2]
GeO_2	White powder in colourless liquid	GeO_2, [Hbet][NTf_2]
In_2O_3	White powder in colourless liquid	In_2O_3, [Hbet][NTf_2]
MgO	Clear, colourless solution	-
MnO	Pale orange, clear paste	Many unidentified reflections
MnO_2	Black powder in colourless liquid	MnO_2
MoO_3	White powder in colourless liquid	MoO_3, [Hbet][NTf_2]
Nb_2O_5	White powder in colourless liquid	Nb_2O_5, [Hbet][NTf_2]
NiO	Green powder in colourless liquid	NiO, [$(Hbet)_3$(bet)][NTf_2]$_3$
PbO	Clear, colourless solution	-
PbO_2	Clear, colourless solution	-
ReO_3	Red crystals in colourless liquid	ReO_3
Sb_2O_3	White powder in colourless liquid	Sb_2O_3, [$(Hbet)_3$(bet)][NTf_2]$_3$
SnO	White powder in brown liquid	SnO, [Hbet][NTf_2]

SrO	Pale orange, opaque paste	Many unidentified reflections
ThO_2	Black powder in colourless liquid	ThO_2, few unidentified reflections (present in reagent)
TiO_2	White powder in colourless liquid	TiO_2, [Hbet][NTf_2]
V_2O_3	Small blue-green crystals in colourless liquid	Several unidentified reflections
V_2O_5	Yellow powder in brown liquid	V_2O_5, many unidentified reflections
WO_3	Yellow powder in colourless liquid	WO_3
ZnO	First colourless solution gels to white paste	Many unidentified reflections

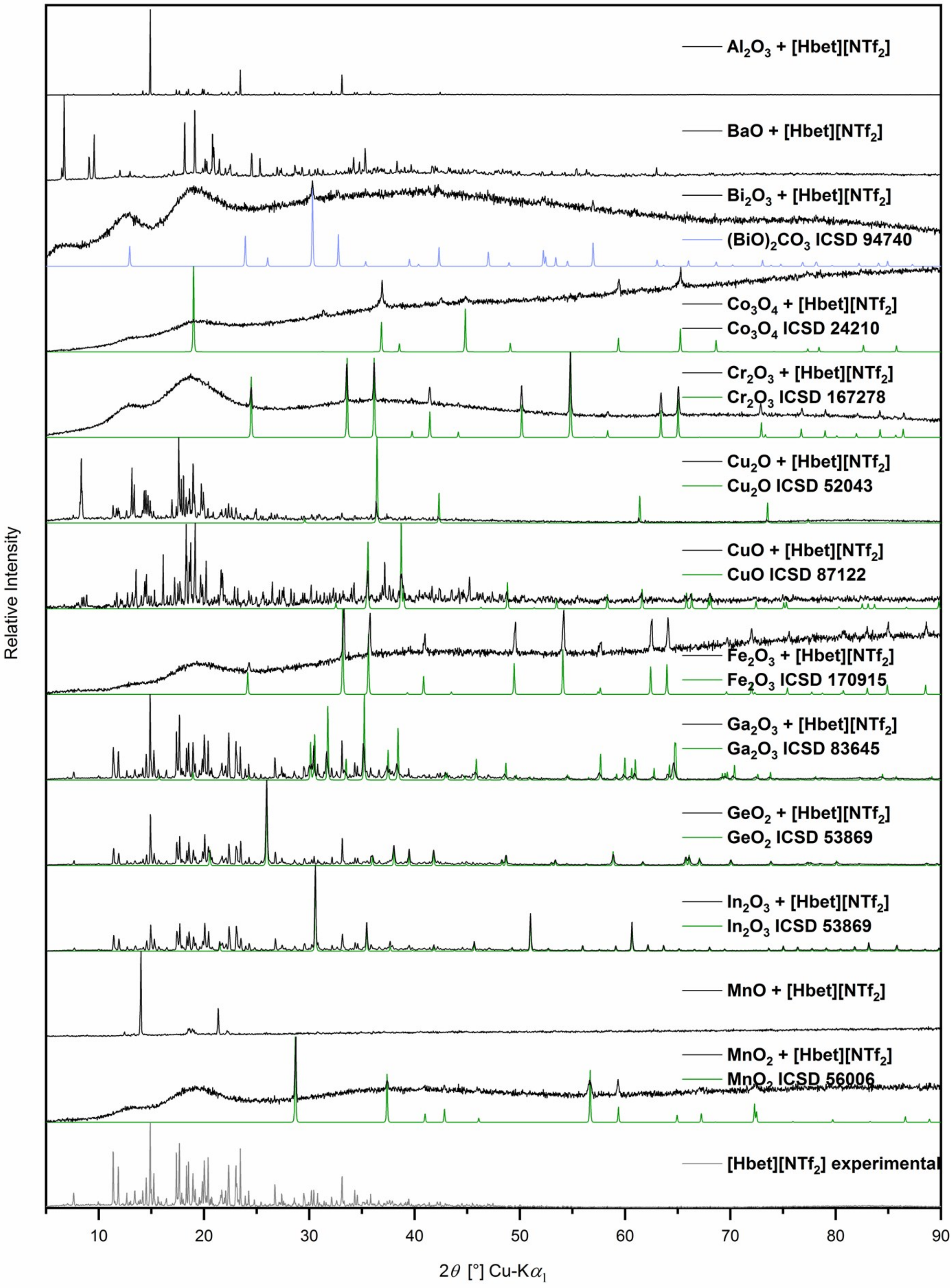

Fig. S4 Experimental diffractograms of the samples Al_2O_3, BaO, Bi_2O_3, Co_3O_4, Cr_2O_3. Cu_2O, CuO, Fe_2O_3, Ga_2O_3, GeO_2, In_2O_3, MnO and MnO_2 + [Hbet][NTf$_2$] (black) in the range $5° \leq 2\theta \leq 90°$ compared to the reflection patterns of the respective metal oxide if present (green) and $(BiO)_2CO_3$ (violet) simulated from single crystal data as well as the experimental reagent pattern of [Hbet][NTf$_2$] (grey). Unidentified reflections in predominantly or completely unidentified patterns (BaO, Cu_2O, CuO, MnO) are not marked as such.

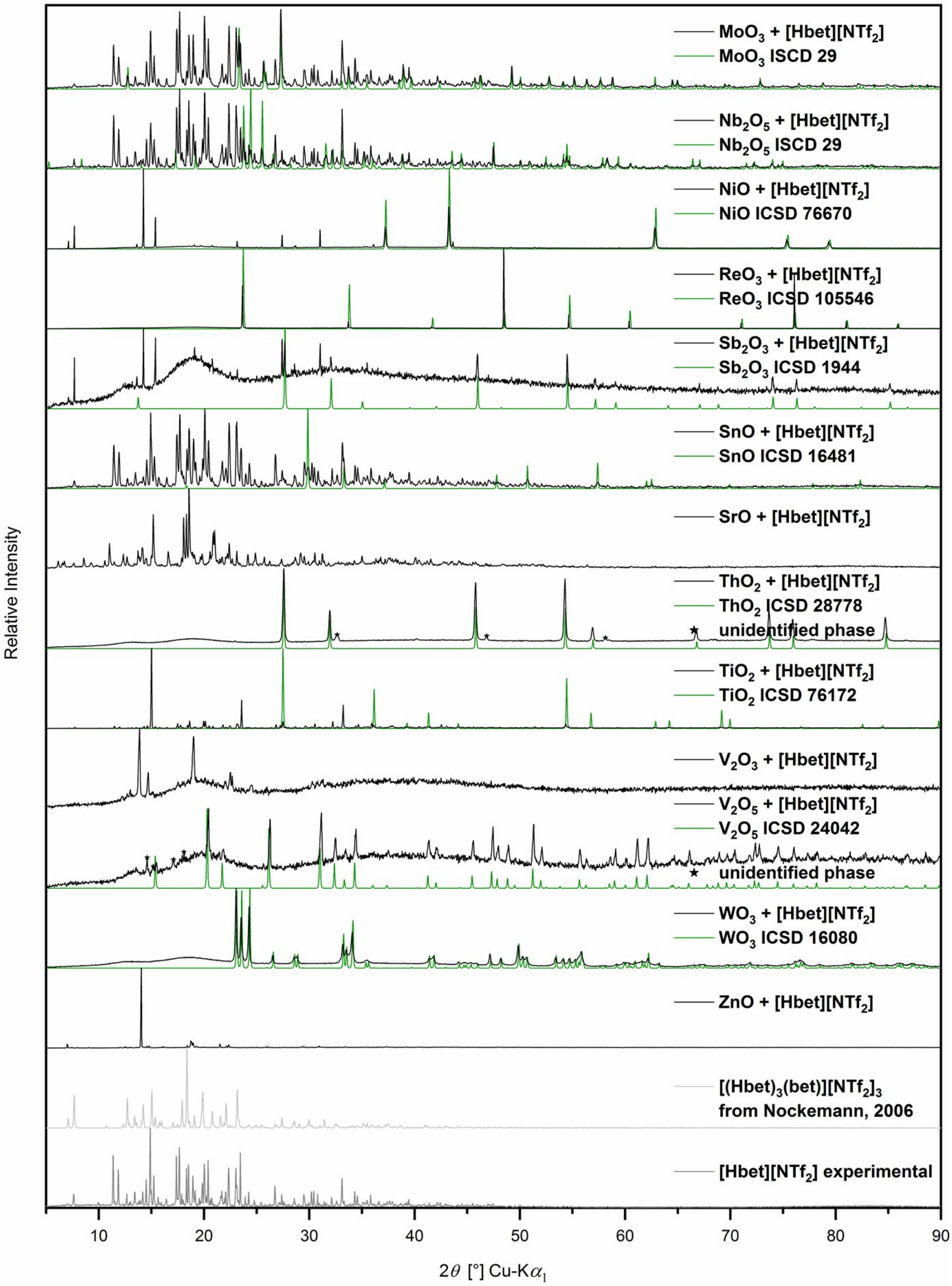

Fig. S5 Experimental diffractograms of the samples MoO_3, Nb_2O_5, NiO, ReO_3, Sb_2O_3, SnO, SrO, ThO_2, TiO_2, V_2O_3, V_2O_5, WO_3 and ZnO + [Hbet][NTf_2] (black) in the range $5° \leq 2\theta \leq 90°$ compared to the reflection patterns of the respective metal oxide if present simulated from single crystal data (green) as well as the experimental reagent pattern of [Hbet][NTf_2] (dark grey) and the pattern of [$(Hbet)_3$(bet)][NTf_2]$_3$ (light gray) simulated from single crystal data.[1] Unidentified reflections in completely unidentified patterns (SrO, V_2O_3, ZnO) are not marked as such.

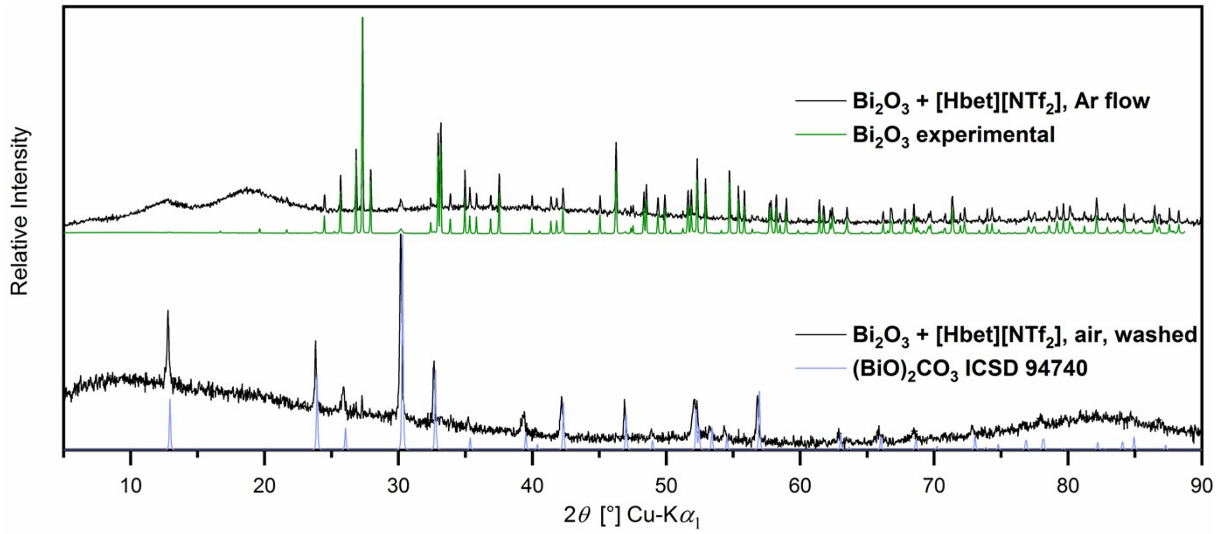

Fig. S6 Experimental diffractograms of the samples Bi_2O_3 + [Hbet][NTf$_2$] reacted in argon flow (top) and on air before washing with acetone (bottom) compared to the experimental pattern of the reagent Bi_2O_3 (green) and the $(BiO)_2CO_3$ pattern calculated from single crystal data (violet) in the range $5° \leq 2\theta \leq 90°$.

Lattice energies and *U*/*x* values

Table S2 Data for the calculation of the lattice energy *U* by the Born-Haber cycle and of the *U*/*x* value. Furthermore, the binding energy of O_2 B = 498.34 kJ/mol and the electron affinities of O EA_1 = 141 kJ/mol and EA_2 = –844 kJ/mol were used. ΔH_f values were obtained from *Thermochemical Data of Pure Substances*,[2] ΔH_s, ΔH_m, ΔH_v and B from *Lange's Handbook of Chemistry*[3] and I_i and EA_i values from the *NIST* online database.[4]

Oxide	*x*	ΔH_f [kJ/mol]	ΔH_s [kJ/mol]	ΔH_m [kJ/mol]	ΔH_v [kJ/mol]	$\sum I_i$ [kJ/mol]]	*U* [kJ/mol]	*U*/*x* [kJ/mol]
Al_2O_3	2	–1676	326	–	–	2394	15464	7732
BaO	1	–554	–	7.12	140.3	1468	3121	3121
Bi_2O_3	2	–574	–	11.30	151	4781	13318	6659
CaO	1	–635	–	8.45	154.7	1735	3486	3486
Co_3O_4	3	–910	424	–	–	4025	18067	6022
Cr_2O_3	2	–1140	397	–	–	5231	15252	7626
Cu_2O	2	–171	337.7	–	–	745	3290	1645
CuO	1	–156	337.7	–	–	2703	4150	4150
Fe_2O_3	2	–824	415.5	–	–	5283	15078	7539
Ga_2O_3	2	–1089	–	5.59	254	5523	15511	7756
GeO_2	1	–580	–	36.94	334	9997	12852	12852
In_2O_3	2	–926	243.1	–	–	5085	14439	7220
MgO	1	–601	147	–	–	2188	3889	3889
MnO	1	–385	–	12.9	221	2226	3798	3798
MnO_2	1	–520	–	12.9	221	10416	13075	13075
MoO_3	1	–745	664	–	–	20643	24909	24909
Nb_2O_5	2	–1900	726	–	–	12958	34030	17015
NiO	1	–240	–	17.48	377.5	2490	4077	4077
PbO	1	–218	195.2	–	–	2166	3532	3532
PbO_2	1	–274	195.2	–	–	9332	11706	11706
ReO_3	1	–589	779	–	–	20207	24433	24433
Sb_2O_3	2	–720	–	19.87	193.43	4878	13760	6880
SnO	1	–286	–	7.03	296.1	2120	3662	3662
SrO	1	–592	164.0	–	–	1614	3322	3322
ThO_2	1	–1226	–	13.81	514	6308	9967	9967
TiO_2	1	–945	469	–	–	8796	12114	12114
V_2O_3	2	–1219	516	–	–	4891	14890	7445
V_2O_5	2	–1551	516	–	–	15696	38738	19369
WO_3	1	–843	851	–	–	19761	24312	24312
ZnO	1	–350	–	7.32	123.6	2640	4074	4074

The compound $[Cu_2(bet)_4(NTf_2)_2][NTf_2]_2$

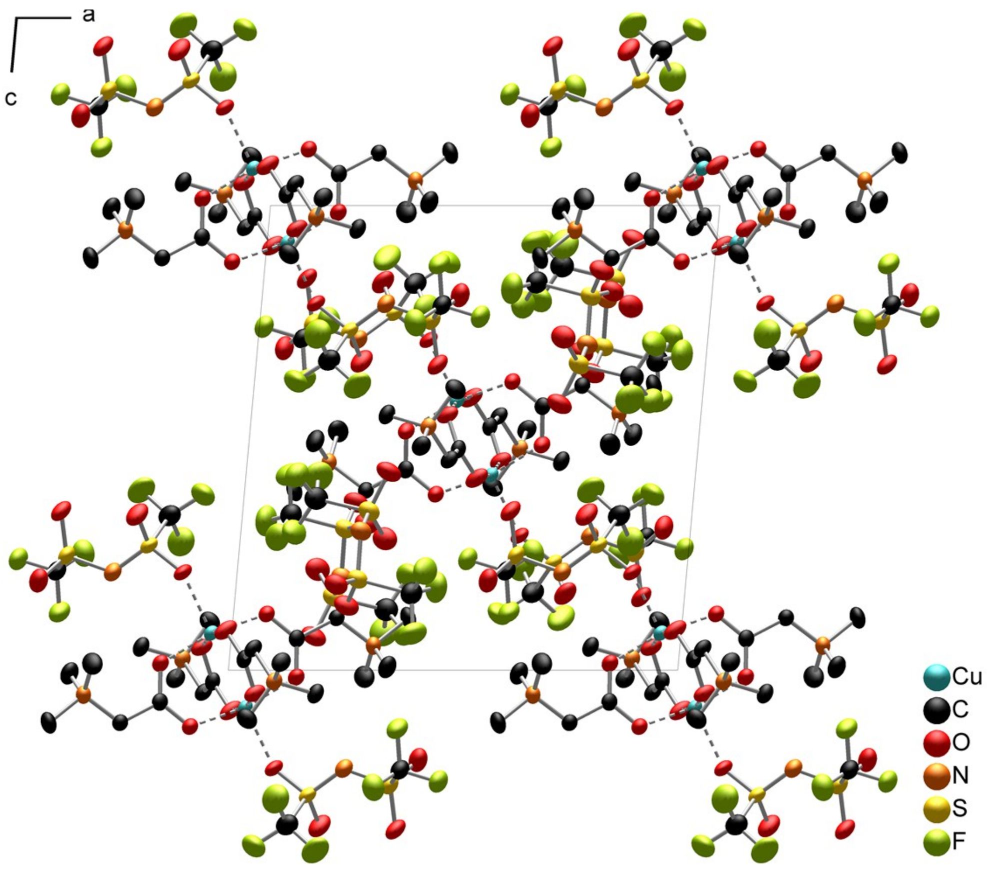

Fig. S7 Crystal structure of $[Cu_2(bet)_4(NTf_2)_2][NTf_2]_2$. Coordinative interactions are marked as dotted lines. The ellipsoids enclose 70 % of the probability density of the atoms at 100 K. H atoms are omitted for clarity.

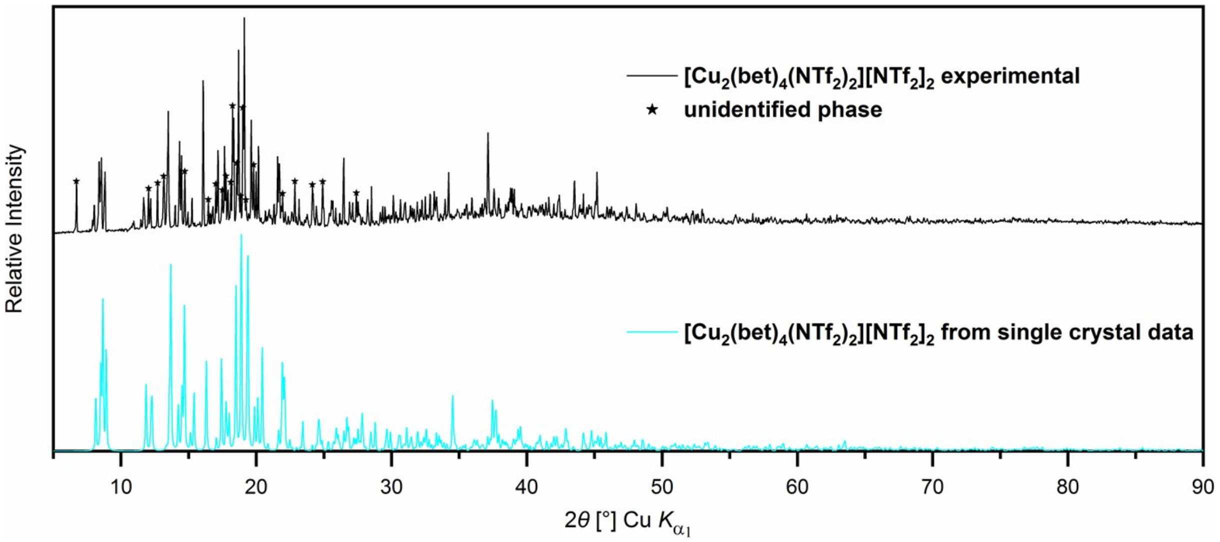

Fig. S8 Experimental diffractogram of $[Cu_2(bet)_4(NTf_2)_2][NTf_2]_2$ after washing with acetone (black) compared to the pattern simulated from single crystal data of $[Cu_2(bet)_4(NTf_2)_2][NTf_2]_2$ (blue) in the range $5° \leq 2\theta \leq 90°$.

Assignment of IR bands of [Hbet][NTf_2]

Table S3 Positions and proposed assignment of the bands observed in the FTIR spectrum of [Hbet][NTf_2] in the range 500 $cm^{-1} \leq \tilde{\nu} \leq 4000\ cm^{-1}$. The symbols have their usual meaning: ν stretching, δ bending, γ out of plane bending or wagging, $_s$ symmetric, $_{as}$ asymmetric. Assignment with the aid of references 5–8.

**IR vibration of [Hbet][NTf_2] [cm^{-1}]**	**Proposed assignment**
3301	ν_{as} OH
3053	ν_s CH (CH_3)
2999	ν_{as} CH (CH_3)
2966	ν CH (CH_2)
1770	ν_{as} COO
1496	ν_{as} HCH (CH_3)
1479	δ_{as} CH_3
1424	δ_s HCH (CH_3-N)
1350	ν_{as} SO_2
1331	δ NCH
1180	ν CF_3
1142	ν_s SO_2
1050	ν_{as} SN
994	ν_{as} C_3N
955	δ CCN
931	δ CNC
883	ν CC
795	ν_s SN
766	ν CS
743	δ_s CF_3
676	ν CN
610	δ CSN
572	γ CH
518	γ CH

^{1}H NMR spectra

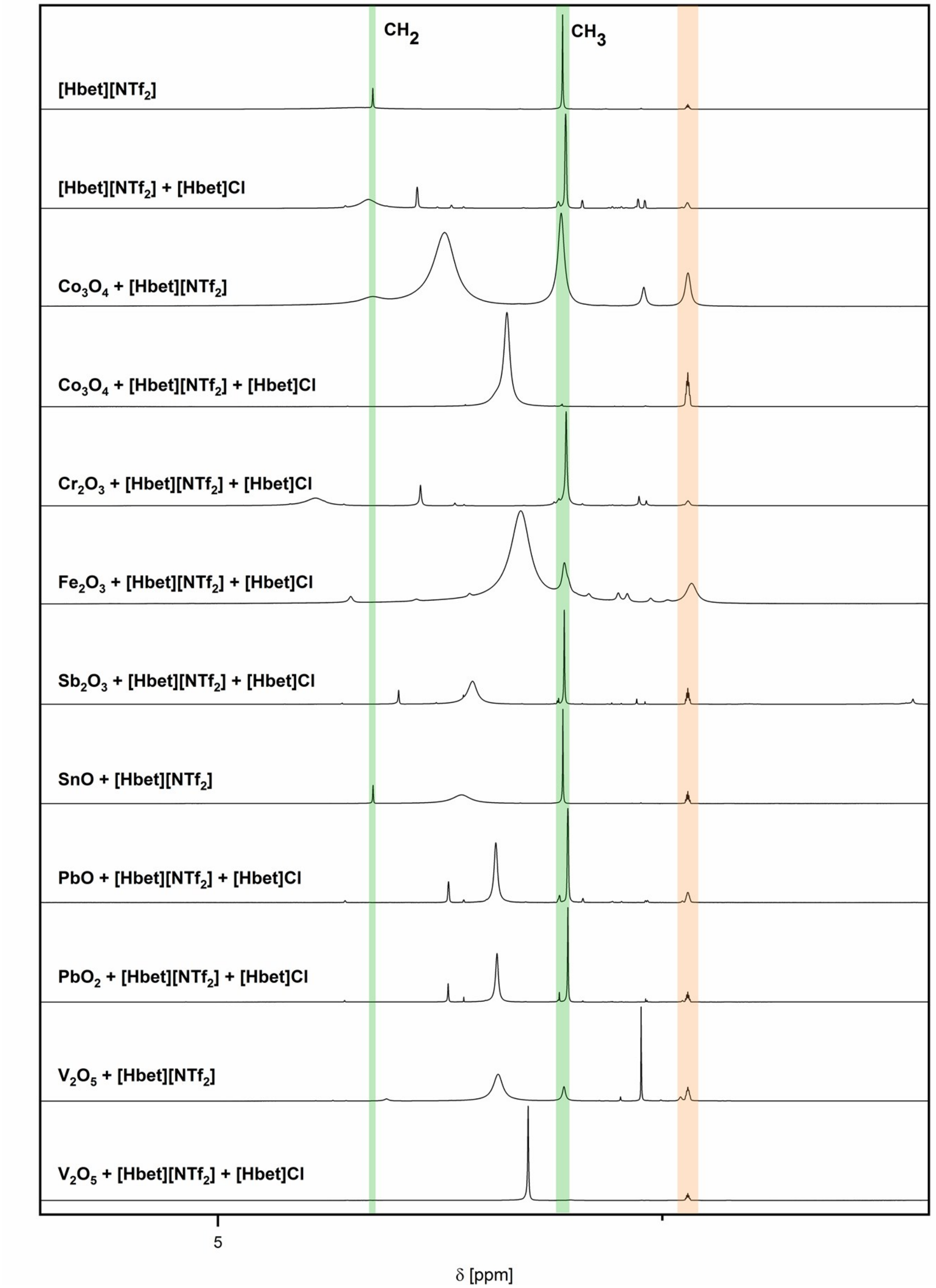

Fig S9 1H NMR spectra of [Hbet][NTf_2], the heated mixture of [Hbet][NTf_2] and [Hbet]Cl as well as several samples of metal oxide mixtures in the range 1 ppm ≤ δ ≤ 6 ppm, where all signals occur. Highlighted in green are the signals of CH_3 (3.1 ppm) and CH_2 (4.1 ppm), orange shading indicates the

signal originating from the solvent DMSO-d_6. No signal is observed for the carboxyl proton of betainium, which is attributed to its low intensity und broadness due to fast exchange processes.[9]

Reaction mixtures in [Hbet][NTf$_2$]-[Hbet]Cl

Table S4 Product appearance and phases identified by pXRD of the reaction mixtures of a metal oxide, [Hbet][NTf$_2$] and [Hbet]Cl. If not stated otherwise, a molar ratio of $n_M : n_{[Hbet][NTf2]} : n_{[Hbet]Cl} = 1 : 2 : 2$ and heating to 175 °C for 24 h was applied.

Oxide	Product appearance	PXRD phases	Varied reaction conditions	Product appearance
Al_2O_3	White powder in brown liquid	No reflections		
BaO	White powder in brown paste	$BaCl_2$, unidentified reflections	$n_{Ba} : n_{[Hbet][NTf2]} : n_{[Hbet]Cl} = 3 : 18 : 1$	Colourless liquid and white solid, identified as $BaCl_2$ by pXRD after washing
Bi_2O_3	White powder in brown liquid	BiOCl, unidentified reflections		
CaO	Paste of brown, orange and colourless crystals	Unidentified reflections	$n_{Ca} : n_{[Hbet][NTf2]} : n_{[Hbet]Cl} = 3 : 18 : 1$	Clear, colourless solution
Co_3O_4	Blue crystals in colourless liquid	Unidentified reflections		
Cr_2O_3	Green powder in colourless liquid	Cr_2O_3		
Cu_2O	White powder in yellow liquid	CuCl		
CuO	Brown solid in brown liquid	Many unidentified reflections	4 h	Green liquid, precipitation of blue, needle-shaped crystals overnight
Fe_2O_3	Red powder in brown liquid	Fe_2O_3, unidentified reflections	$n_{Fe} : n_{[Hbet][NTf2]} : n_{[Hbet]Cl} = 1 : 4 : 1$	Red powder in slighly yellow liquid
Ga_2O_3	White powder in brown paste	Ga_2O_3, unidentified reflections	$n_{Ga} : n_{[Hbet][NTf2]} : n_{[Hbet]Cl} = 1 : 6 : 1$	White powder in colourless liquid, only Ga_2O_3 identified by pXRD
GeO_2	White powder in brown liquid	GeO_2		
In_2O_3	Pale orange paste	In_2O_3, [(Hbet)$_3$(bet)][NTf$_2$]$_3$ unidentified reflections	$n_{In} : n_{[Hbet][NTf2]} : n_{[Hbet]Cl} = 1 : 6 : 1$	Fine, yellow powder in colourless liquid, only In_2O_3 identified by pXRD
MgO	Slightly brown solid	Many unidentified reflections	$n_{Mg} : n_{[Hbet][NTf2]} : n_{[Hbet]Cl} = 3 : 18 : 1$	Clear, colourless solution
MnO	Light brown paste	Many unidentified reflections	$n_{Mn} : n_{[Hbet][NTf2]} : n_{[Hbet]Cl} = 3 : 18 : 1$	Clear, light orange solution
MnO_2	Colourless crystals in brown liquid	Unidentified reflections		
MoO_3	Brown, hard resin-like substance	MoO_3, unidentified reflections		
Nb_2O_5	white powder in brown liquid	Nb_2O_5, unidentified reflections	$n_{Nb} : n_{[Hbet][NTf2]} : n_{[Hbet]Cl} = 1 : 6 : 1$	White powder in colourless liquid, Nb_2O_5 identified by

				pXRD
NiO	Green solid (washing with acetone yields green liquid and hygroscopic yellow powder transforming to green liquid on air)	NiO, unidentified reflections		
PbO	White powder in light brown liquid	Unidentified pattern	n_{Pb} : $n_{[Hbet][NTf2]}$: $n_{[Hbet]Cl}$ = 1 : 4 : 1	White powder in orange solution
PbO_2	White powder in brown liquid	Unidentified pattern		
ReO_3	Red crystals in brown liquid	ReO_3		
Sb_2O_3	white powder in brown liquid	$[(Hbet)_3(bet)][NTf_2]_3$ unidentified reflections		
SnO	Brown paste	No reflections		
SrO	Clear, brown liquid	-		
ThO_2	White powder in brown solution	ThO_2	n_{Th} : $n_{[Hbet][NTf2]}$: $n_{[Hbet]Cl}$ = 1 : 12: 2	White powder and a few black particles in yellow liquid
TiO_2	white powder in brown liquid	TiO_2, $[(Hbet)_3(bet)][NTf_2]_3$		
V_2O_3	Fine, black powder in brown paste	V_2O_3	n_{V} : $n_{[Hbet][NTf2]}$: $n_{[Hbet]Cl}$ = 1 : 6 : 1	Few black particles in grey liquid, no pXRD signals
V_2O_5	Black powder in dark green paste	No reflections		
WO_3	Yellow powder in brown liquid	WO_3		
ZnO	Clear, brown liquid	-		

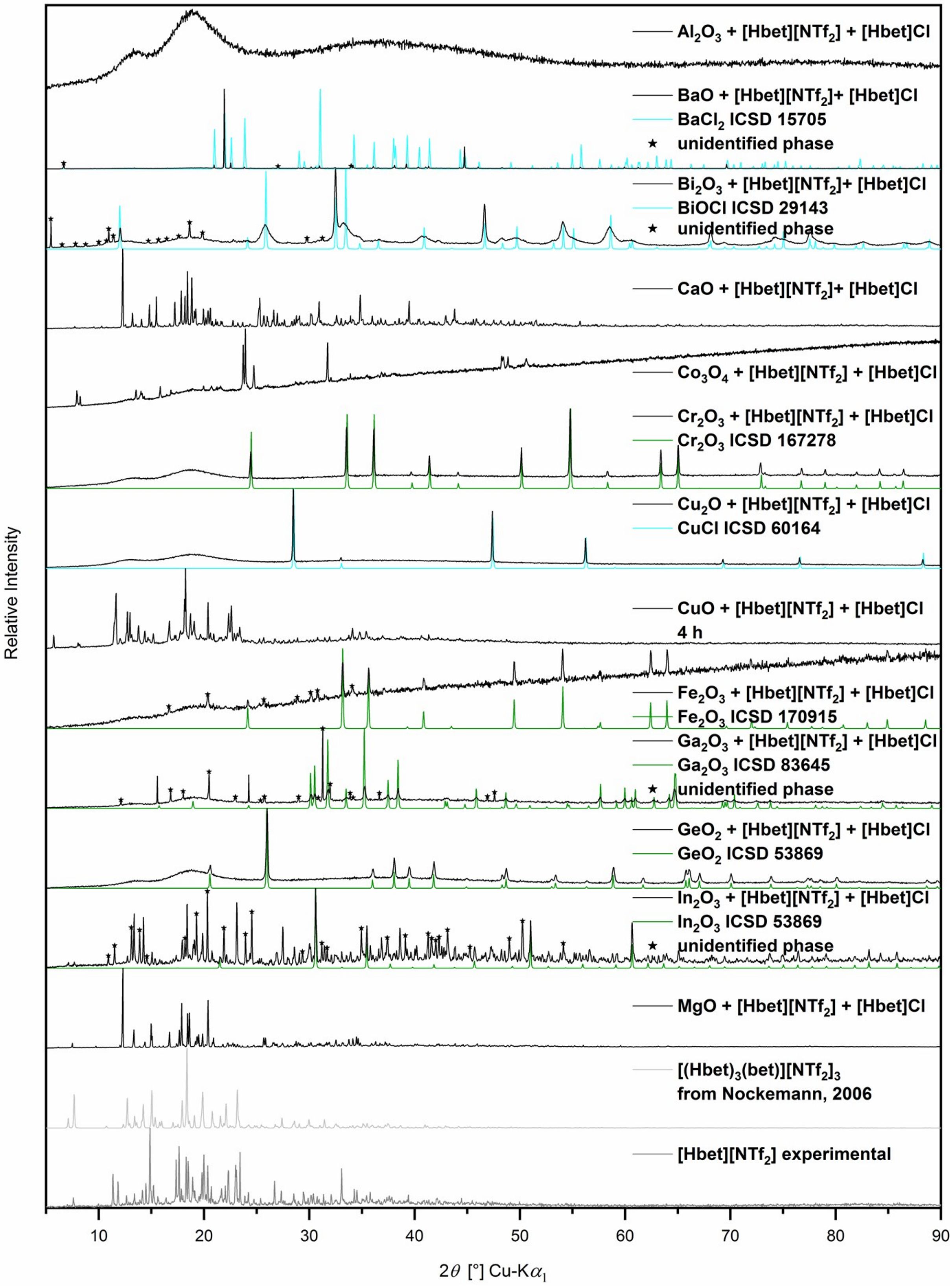

Fig. S10 Experimental diffractograms of the samples Al_2O_3, BaO, Bi_2O_3. CaO, Co_3O_4, Cr_2O_3, Cu_2O, CuO, Fe_2O_3, Ga_2O_3, GeO_2 and In_2O_3 + [Hbet][NTf_2] + [Hbet]Cl (black) in the range $5° \leq 2\theta \leq 90°$ compared to the reflection patterns of the respective metal oxide (green) or metal chloride/oxide chloride (blue) if present simulated from single crystal data as well as the experimental reagent pattern of [Hbet][NTf_2] (dark grey) and the pattern of [$(Hbet)_3$(bet)][NTf_2]$_3$ simulated from single crystal data.[1] Unidentified reflections in completely unidentified patterns (CaO, Co_3O_4, CuO, MgO) are not marked as such.

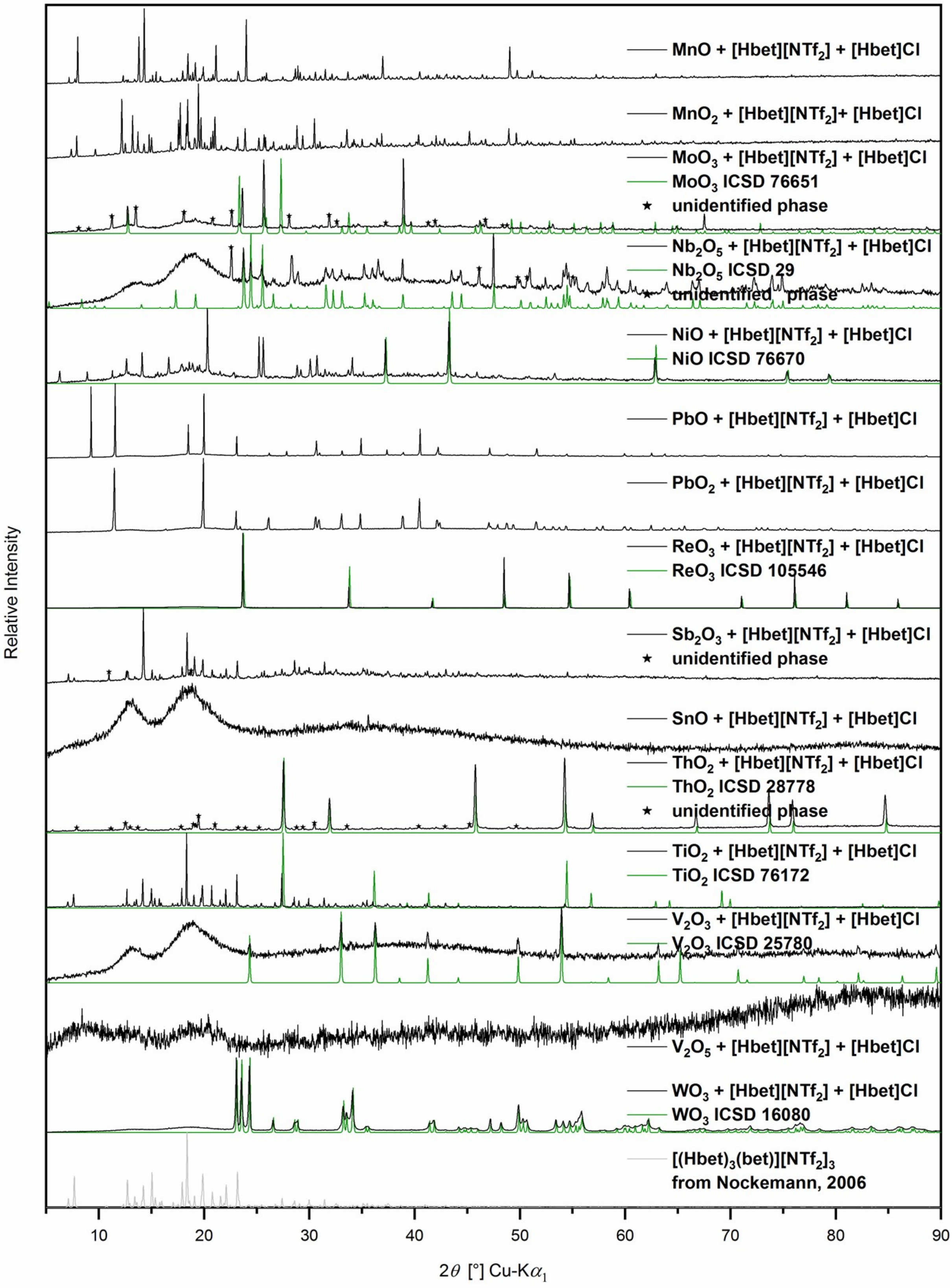

Fig. S11 Experimental diffractograms of the samples MnO, MnO_2, MoO_3, Nb_2O_5, NiO, PbO, PbO_2, ReO_3, Sb_2O_3, SnO, ThO_2, TiO_2, V_2O_3, V_2O_5 and WO_3 + [Hbet][NTf_2] + [Hbet]Cl (black) in the range $5° \leq 2\theta \leq 90°$ compared to the reflection patterns of the respective metal oxide if present simulated from single crystal data (green) as well as the pattern of [$(Hbet)_3$(bet)][NTf_2]$_3$ simulated from single crystal data.[1] Unidentified reflections in predominantly or completely unidentified patterns (MnO, MnO_2, NiO, PbO, PbO_2) are not marked as such.

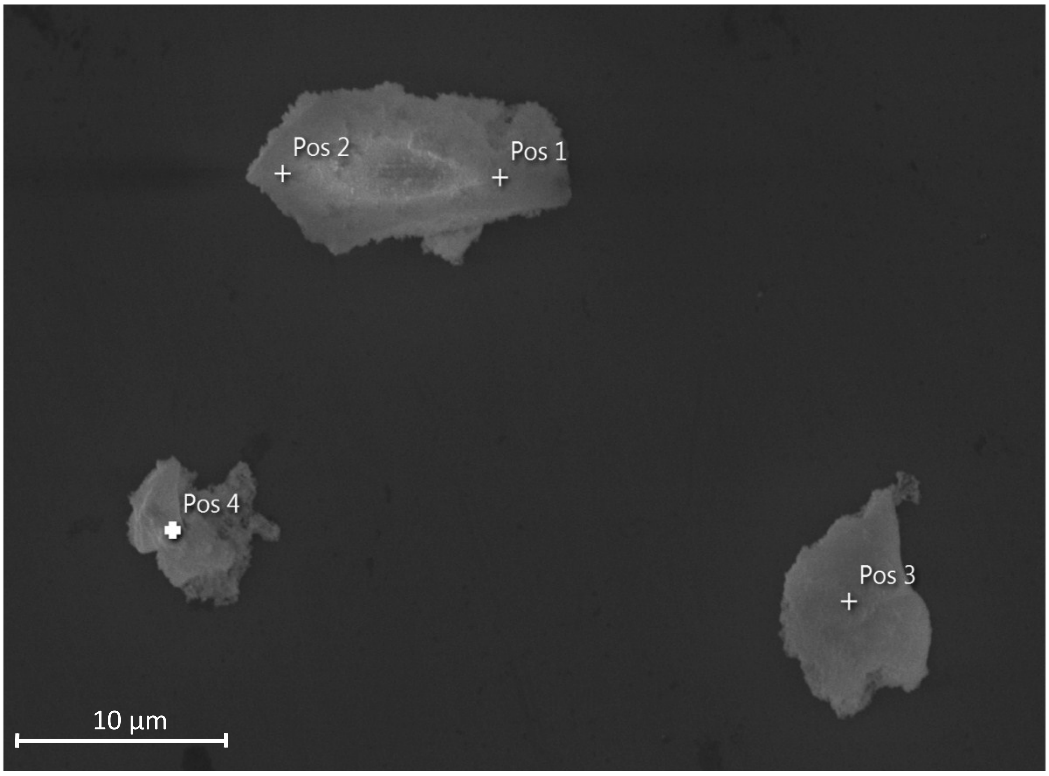

EDX of the sample ThO_2 + [Hbet][NTf_2] + [Hbet]Cl

Fig S12 SEM image of the white powder of the sample ThO_2 + [Hbet][NTf_2] + [Hbet]Cl (n_{Th} : $n_{[Hbet][NTf2]}$: $n_{[Hbet]Cl}$ = 1 :12 : 2) with EDX measuring points indicated.

Table S5 Overview of the results of the EDX measurement. Besides C, O and Th, also La and Ta were detected. However, as their amounts were below the detection level, no assumption of the presence of small quantities of these elements can be made.

**Element**	**EDX composition**				
	Pos. 1	Pos. 2	Pos. 3	Pos. 4	Average
C	52 %	50 %	56 %	50 %	52 %
O	35 %	39 %	36 %	36 %	36 %
Th	13 %	11 %	9 %	13 %	11 %

References

1 P. Nockemann, B. Thijs, S. Pittois, J. Thoen, C. Glorieux, K. Van Hecke, L. Van Meervelt, B. Kirchner and K. Binnemans, *J. Phys. Chem. B*, 2006, **110**, 20978–20992.
2 I. Barin, *Thermochemical Data of Pure Substances*, VCH, Weinheim, 3rd edn., 1995.
3 J. G. Speight, *Lange's handbook of chemistry*, McGraw-Hill, New York, 16th edn., 2005.
4 A. Kramida, Y. Ralchenko and J. Reader, *NIST Atomic Spectra Database 5.6.1*, National Institute of Standards and Technology, Gaithersburg, 2018.
5 M. Viertorinne, J. Valkonen, I. Pitkänen, M. Mathlouthi and J. Nurmi, *J. Mol. Struct.*, 1999, **477**, 23–29.
6 K. Hanke, M. Kaufmann, G. Schwaab, M. Havenith, C. T. Wolke, O. Gorlova, M. A. Johnson, B. P. Kar, W. Sander and E. Sanchez-Garcia, *Phys. Chem. Chem. Phys.*, 2015, **17**, 8518–8529.
7 M. M. Ilczyszyn and M. Ilczyszyn, *J. Raman Spectrosc.*, 2003, **34**, 693–704.
8 M. Szafran, A. Katrusiak, Z. Dega-Szafran and I. Kowalczyk, *J. Mol. Struct.*, 2013, **1031**, 49–55.

9 J. Dimitric-Markovic, U. Mioc, J. Baranac and Z. Nedic, *Journal of the Serbian Chemical Society*, 2001, **66**, 451–462.
